# Matching TGF‐β1 Activation With Stress‐Responsive Charge‐Reversal Hydrogel Microspheres for the Treatment of Osteoarthritis

**DOI:** 10.1002/EXP.20250363

**Published:** 2026-06-09

**Authors:** Feng Lin, Yiwen Xu, Lei Xiang, Yueliang Zhu, Yihan Li, Yaping Zhuang, Zengjie Zhang, Jiayu Chen, Heemin Kang, Ning Zhang, Xiaohua Yu, Wenguo Cui

**Affiliations:** ^1^ Department of Orthopaedic Surgery Key Laboratory of Motor System Disease Research and Precision Therapy of Zhejiang Province The Second Affiliated Hospital Zhejiang University School of Medicine Orthopedics Research Institute of Zhejiang University Clinical Research Center of Motor System Disease of Zhejiang Province Hangzhou P. R. China; ^2^ Department of Orthopaedics Shanghai Key Laboratory For Prevention and Treatment of Bone and Joint Diseases Shanghai Institute of Traumatology and Orthopaedics; Ruijin Hospital Shanghai Jiao Tong University School of Medicine Shanghai P. R. China; ^3^ Department of Materials Science and Engineering Korea University Seoul Republic of Korea

**Keywords:** charge reversal, hydrogel microspheres, osteoarthritis, stress‐responsive hydrogels, transforming growth factor‐β1

## Abstract

The abnormal activation of transforming growth factor‐β1 (TGF‐β1) is closely associated with the occurrence and progression of diseases. However, how to make the regulatory means match the pathological mechanical microenvironment‐dependent activation of TGF‐β1 to promote lesion repair still faces challenges. In this study, based on force‐charge conversion technology, we developed the stress‐responsive charge‐reversal hydrogel microsphere system. Weakly cross‐linked disulfide bonds in the microfluidic‐synthesized microspheres break under mechanical stress, interacting with charge‐reversal messenger to induce nanoparticle charge reversal (negative to positive). This mode is in line with the activation mechanism of TGF‐β1 in the mechanical microenvironment, which can match the appropriate regulatory intensity and thus achieve high efficiency and low toxicity in OA treatment. Experimental results show that this hydrogel microsphere system has a 41.96% improvement in the repair effect of OA cartilage lesions compared to traditional microspheres, and more importantly, the impact on healthy cartilage is reduced by 96.89%. This study provides a new idea for the development of biomaterial regulatory systems that match the pathological microenvironment.

## Graphical abstract

Based on the interaction between the weakly cross‐linked disulfide bonds that break under stress and charge‐reversal messenger molecules, we herein achieved nanoparticle charge reversal under stress and constructed a stress‐responsive charge‐reversal hydrogel microsphere system using microfluidic technology. Therefore, we can achieve the matching of regulatory methods with the pathological mechanical microenvironment‐dependent TGF‐β1 activation for the treatment of OA.

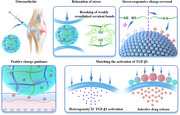

## 1 Introduction

Transforming growth factor‐β1 (TGF‐β1) is a critical factor in biological research, particularly in the regulation of cell function and disease progression [1, 2, 3, 4]. There is a close interaction between TGF‐β1 activation and mechanical stress, which plays a pivotal role in tissue homeostasis, repair, fibrosis, and disease progression [5, 6, 7]. Therefore, matching the pathomechanical microenvironment‐dependent activation of TGF‐β1 remains a significant challenge for the design of biomaterials aimed at lesion tissue repair.

In the pathological microenvironment of osteoarthritis (OA), persistent abnormal mechanical stress causes chondrocytes to regulate their metabolic activities and cytokine expression in response to changes in the extracellular mechanical stress environment [8, 9]. Abnormally synthesized transforming growth factor‐β1 (TGF‐β1) induces cellular redox imbalance by increasing the production of ROS in the mitochondrial respiratory chain of various cell populations, including chondrocytes, leading to cell apoptosis, senescence, and fibrosis gene expression [10, 11]. More importantly, Zhen et al. [12] reported that TGF‐β1 is deposited in the articular cartilage matrix after abnormal synthesis and secretion by chondrocytes. Under stress conditions, the deposited TGF‐β1 can be induced to transform into active form, and the degree of mechanical stress is positively correlated with the activation of TGF‐β1.

Therefore, researchers have developed multifunctional hydrogel microsphere systems to release drugs into the deep layers of cartilage tissue, thereby alleviating the pathological changes of chondrocytes under abnormal mechanical stress [13, 14, 15, 16]. For example, Lin et al. [17] developed a charge‐guided hydrogel microsphere system that not only effectively relives stress overload but also inhibits the negative effects of activated TGF‐β1 by guiding positively charged nanoparticles to penetrate deep into the cartilage matrix through positive and negative charge interactions for the targeted release of drugs to chondrocytes. Although the charge‐guided hydrogel microsphere system can penetrate deep into the cartilage matrix and target deep chondrocytes to release drugs, effectively alleviating the pathological changes of chondrocytes caused by excessive activation of TGF‐β1, the arc‐shaped surface of articular cartilage and the deviation of the lower limb force line often lead to uneven stress distribution on the cartilage surface, resulting in different degrees of TGF‐β1 activation dependent on the mechanical microenvironment in different areas. Therefore, non‐discriminatory regulatory methods will inhibit the activation of TGF‐β1 with physiological functions, causing significant side effects. To solve the above problems, it is necessary to construct a hydrogel microsphere system that targets deep chondrocytes, matches the pathological mechanical microenvironment and TGF‐β1 activation, so as to match the appropriate regulatory intensity for the pathological mechanical microenvironment‐dependent activation of TGF‐β1 and achieve efficient and low‐toxicity treatment of OA.

To construct biomaterials with matching microenvironmental characteristic factors of lesions (such as mechanical stress, pH, temperature, ROS, etc.), researchers have introduced the concept of charge‐reversal. For example, researchers have leveraged the specific reaction between *N*‐(2‐hydroxythyl)‐4‐azido‐1,8‐naphthimide (Nap‐N_3_) and hydrogen sulfide (H_2_S) to develop Nap‐N_3_‐loaded nano micelles with a charge‐reversal function. Upon triggering by H_2_S in tumor lesions, Nap‐N_3_ is converted to Nap‐NH_3_^+^, resulting in positively charged nano micelles, which increase their uptake by the lesions [18]. Finally, the biomaterial system can release high concentrations of drugs in the center of the lesion and low concentrations of drugs or no drugs in the surrounding area, thereby greatly reducing the side effects of drugs and improving their efficacy. Therefore, by integrating the concept of charge‐reversal and the stress‐responsive function into the charge‐guided hydrogel microsphere system, the internal drug delivery nanoparticles of the hydrogel microspheres can be endowed with positive charges under the trigger of the characteristic factor of cartilage lesions (i.e., stress), thereby enhancing the ability of nanoparticles to penetrate deep into the cartilage matrix and match the activation of TGF‐β1 under the guidance of charges. Most importantly, in healthy cartilage, drug‐loaded nanoparticles in the hydrogel microsphere system are electrically neutral and cannot penetrate deeply into the cartilage matrix, whereas free nanoparticles in the joint cavity are removed by the synovial membrane in a short time, thereby preventing the release of drugs to chondrocytes and protecting the healthy cartilage. Ultimately, achieve the matching of regulatory means with the pathological mechanical microenvironment‐dependent TGF‐β1 activation for the treatment of OA.

**SCHEME 1:**
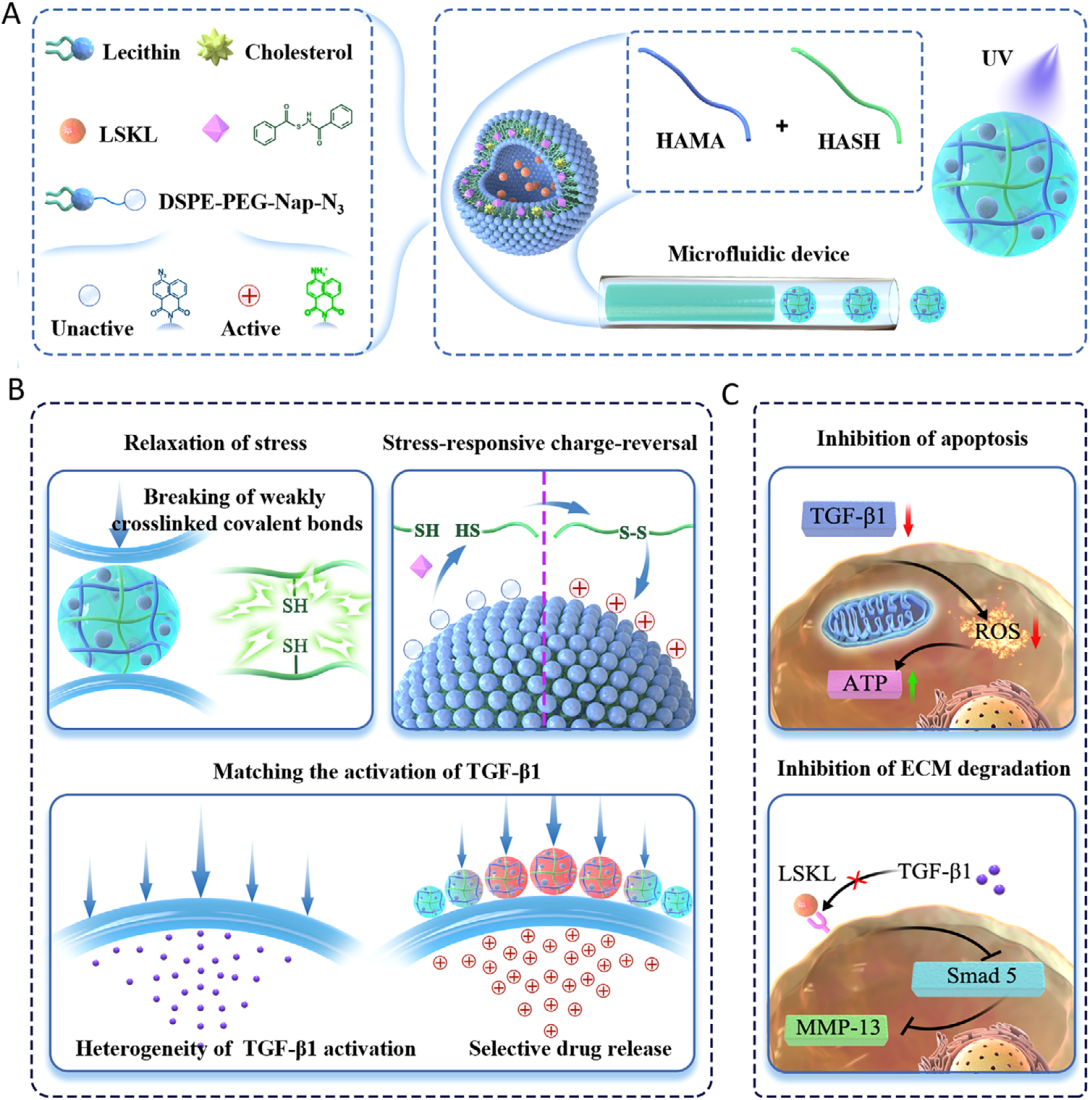
Schematic diagram of the matching TGF‐β1 activation and treatment of osteoarthritis (OA) using the stress‐responsive charge‐reversal hydrogel microsphere system. (A) Construction of a stress‐responsive charge‐reversal hydrogel microsphere system. (B) The hydrogel microsphere system uses the stress‐responsive charge‐reversal mechanism to accurately identify the spatial heterogeneity of cartilage lesions and realize matching TGF‐β1 activation and treatment of OA. (C) The hydrogel microsphere system can effectively improve the efficacy of OA treatment by inhibiting chondrocyte apoptosis and extracellular matrix degradation at the cellular and molecular levels.

In this study, we developed the force‐charge conversion technology. Based on the interaction between the weakly cross‐linked disulfide bonds that break under stress and charge‐reversal messenger molecules, we herein achieved nanoparticle charge reversal under stress and constructed a stress‐responsive charge‐reversal hydrogel microsphere system using microfluidic technology. The hydrogel microspheres achieved bearing lubrication and stress relaxation in the articular cavity were realized, and the stress‐responsive charge‐reversal was realized in the stress‐overloaded cartilage lesions. Under the guidance of the positive charge, the drug‐loaded nanoparticles in the hydrogel microspheres significantly improved the ability to penetrate into the cartilage matrix and target deep chondrocytes and match the activation of TGF‐β1. Finally, according to the different activation states of TGF‐β1 in the mechanical microenvironment, the appropriate regulatory intensity can be matched to achieve high efficiency and low toxicity in the treatment of OA (Scheme 1). First, neutral nanoliposomes with a Nap‐N_3_ structure and N‐(benzoylthio)benzamide as a trigger molecule were prepared via thin‐film hydration. Methylacrylated (HAMA) and thiolated hyaluronic acid (HASH) were synthesized to form a dual‐crosslinked hydrogel network via UV‐induced radical and disulfide bonding. Using microfluidics, hydrogel microspheres loaded with these nanoliposomes were fabricated. The spherical microstructure provides lubrication, reduces friction‐induced cartilage damage, and dissipates mechanical stress through reversible breakage of disulfide bonds in overloaded areas. Upon stress, cleaved disulfide bonds release free thiols, which trigger a reaction between the Nap‐N_3_ group and the messenger molecule, inducing charge reversal of the nanoliposomes from neutral to positive. This promotes deep penetration into the negatively charged cartilage matrix, enabling targeted delivery of therapeutics near chondrocytes. The system effectively suppresses aberrant TGF‐β1 signaling, inhibits chondrocyte apoptosis, maintains extracellular matrix homeostasis, and enhances OA treatment efficacy. Ultimately, this system inhibits chondrocyte apoptosis, maintains the balance of extracellular matrix synthesis and degradation, and improves the efficacy of OA treatment. This study provides a new idea for the development of biomaterial regulatory systems that match the pathological microenvironment.

## 2 Results and Discussion

### 2.1 Characterization and Function of the Stress‐Responsive Charge‐Reversal Hydrogel System

**FIGURE 1:**
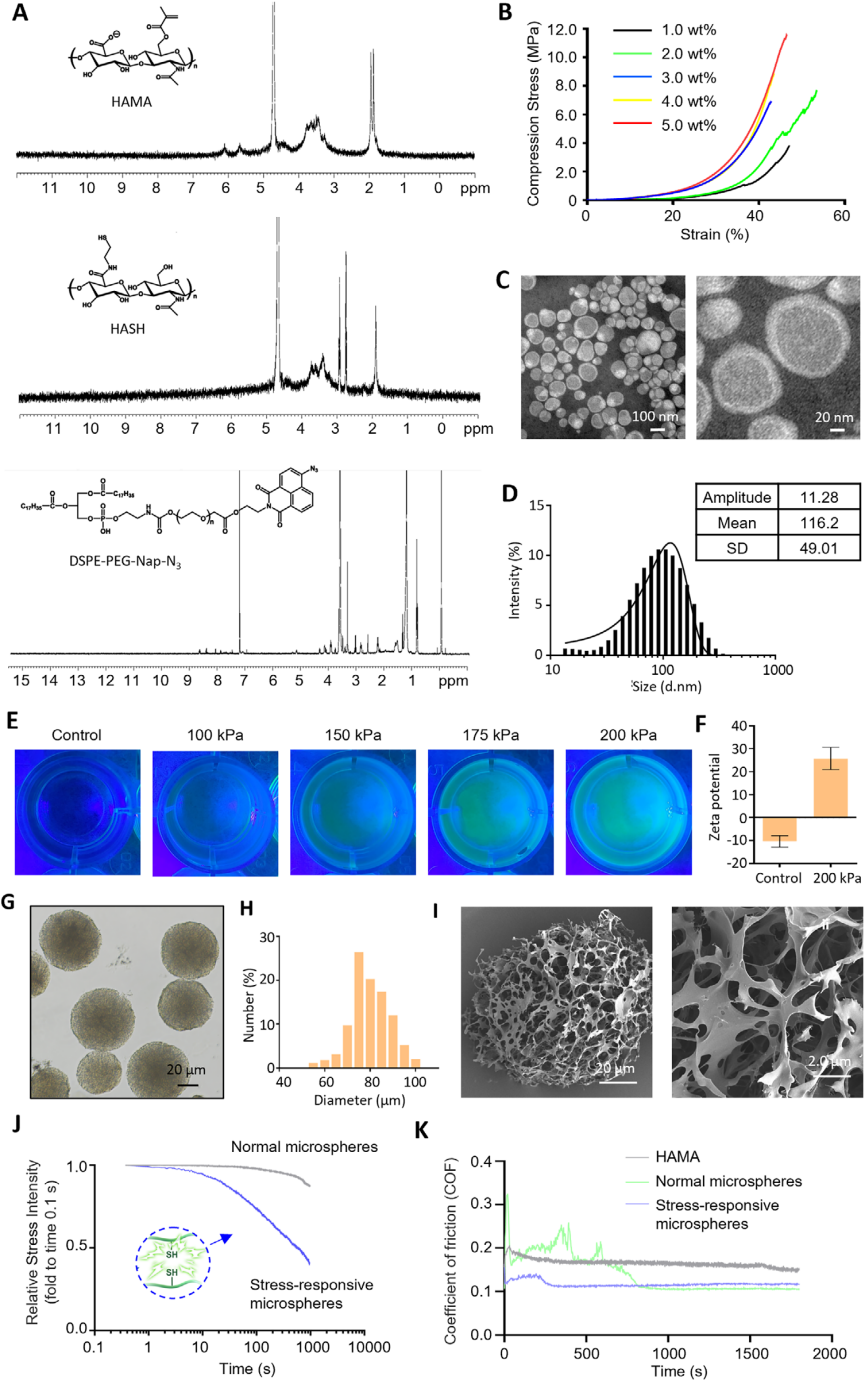
Construction and functional verification of the stress‐responsive charge‐reversal hydrogel microsphere system. (A) ^1^H NMR results of HAMA, HASH, and DSPE‐PEG‐Nap‐N_3_ polymers. (B) Strain curves of the hydrogel networks at different concentrations under pressure. (C) Morphology of the charge‐reversal nanoliposomes as determined via transmission electron microscopy. (D) Particle size distribution of the charge‐reversal nanoliposomes. (E) Proportion of the nanoliposomes achieving charge reversal (reflected by fluorescence intensity) triggered by different stress intensities. (F) Zeta potential of the nanoliposomes at rest and under stress‐responsive conditions. (G) Morphology of the hydrogel microsphere system as evaluated using an optical microscope. (H) Statistical plot of the particle size of the hydrogel microsphere system. (I) Morphology of the hydrogel microsphere system as observed via scanning electron microscopy. (J) Stress relaxation curve of the hydrogel microsphere system. (K) Friction curve of the hydrogel microsphere system. (HAMA: methylacrylated hyaluronic acid; HASH: thiolated hyaluronic acid).

To fabricate the stress‐responsive charge‐reversal hydrogel system, the HAMA and HASH polymers, which form the hydrogel network, and DSPE‐PEG‐Nap‐N_3_ (Phospholipid‐polyethylene glycol‐*N*‐(2‐hydroxyethyl)‐4‐azido‐1,8‐naphthalimide) the key molecule constituting the charge‐reversal nanoparticles, were successfully synthesized (Figure 1A). Through ^1^H NMR spectroscopy and integral calculation, the grafting rates of HAMA (Figure S1, Supporting Information) and HASH (Figure S2, Supporting Information) were determined to be approximately 33% and 50%, respectively, and the grafting rates of DSPE‐PEG‐Nap‐N_3_ were determined to be approximately 15% (Figure S3, Supporting Information). Next, to optimize the mechanical properties of the hydrogel system, different polymer concentrations were tested. The compression‐strain curve revealed that when the polymer concentration ranged within 1.0–3.0 wt%, the mechanical properties of the hydrogel system significantly improved with increasing polymer concentration. However, when the concentration was increased to 3.0–5.0 wt%, no significant further improvement was observed (Figure 1B). Therefore, 3.0 wt% was chosen as the optimal concentration for the hydrogel system. Subsequently, charge‐reversal nanoliposomes were prepared via thin‐film hydration, with NAP‐N_3_ surface modification and loaded with *N*‐(benzoyl sulfur) benzamide as the messenger molecule. The morphology of the charge‐reversal nanoliposomes was examined via transmission electron microscopy, which revealed a uniform size distribution and a distinct shell‐core structure (Figure 1C). Dynamic light scattering measurements showed that the particle size of the liposomes was 116.2 ± 49.01 nm, as determined through Gaussian curve fitting (Figure 1D).

Afterward, the functionality of the stress‐responsive charge‐reversal hydrogel system was validated. Under excessive stress, the cleavage of weakly cross‐linked disulfide bonds generates free sulfite groups, which then trigger an interaction between the Nap‐N_3_ structure on the surface of the charge‐reversal liposomes and the messenger molecule. This results in a shift from electrically neutral to positively charged secondary nanostructures, while the disulfide bonds in the hydrogel microsphere network are reconstituted (Figure S4, Supporting Information). To verify this process, the fluorescence properties of Nap‐N_3_ were used in a preliminary test of the charge‐reversal function of the nanoliposomes (Figure S5A, Supporting Information). The hydrogel system, loaded with the nanoliposomes, was subjected to varying stress levels. Green fluorescence, excited by a 408‐nm laser, indicated that as the stress level increased, a higher proportion of the nanoliposomes underwent charge reversal and the fluorescence intensity threshold for effective charge reversal was about 150 kPa (Figure 1E). Finally, the nanoliposomes within the hydrogel system were released and their zeta potential was measured (Figure S5B, Supporting Information). The results confirmed that the charge‐reversal nanoliposomes successfully transitioned from a negative to a positive charge under stress (Figure 1F). Therefore, the stress‐responsive charge‐reversal hydrogel system was demonstrated to be both successful and effective.

### 2.2 Characterization and Function of the Hydrogel Microsphere System

Based on the hydrogel system constructed above, the stress‐responsive charge‐reversal hydrogel microsphere system was successfully fabricated by using a microfluidic device (Figure S6, Supporting Information). Through optical microscope observation, the hydrogel microsphere system presented a relatively perfect sphere shape (Figure 1G). Moreover, measurements and statistical analysis of the particle size of the hydrogel microsphere system revealed a uniform distribution of particle sizes concentrated at approximately 80 µm (Figure 1H). Subsequently, under a scanning electron microscope, the hydrogel microsphere system demonstrated a relatively uniform pore structure (Figure 1I). Additionally, the energy spectrum detection showed that the hydrogel microsphere system was rich in phosphorus (P) in addition to conventional carbon (C) and oxygen (O), which confirmed the successful construction of the nanoparticle‐loaded hydrogel microsphere system (Figure S7, Supporting Information). And the drug release ability of the hydrogel microsphere system was also examined (Figure S8, Supporting Information). Subsequently, the biocompatibility of the hydrogel microsphere system was tested. Chondrocytes were co‐cultured with nanoparticles (Figure S9A, Supporting Information) and hydrogel microspheres (Figure S9B, Supporting Information) at different concentrations for 48 h, and the proliferation of chondrocytes was detected using the CCK‐8 kit. The results showed that the hydrogel microsphere system did not negatively affect the proliferation of chondrocytes and had good biocompatibility. Finally, the hydrogel microsphere system involved in the above experiments was co‐cultured with chondrocytes for 48 h at the highest concentration of nanoparticles, and the toxicity of the hydrogel microsphere system was verified by live/dead cell staining (Figure S9C, Supporting Information). Under the fluorescence microscope, most chondrocytes were living cells (green fluorescence), and few were dead cells (red fluorescence). Therefore, the developed hydrogel microsphere system exhibited negligible cytotoxicity and can be used as a medical biomaterial with good biocompatibility.

Subsequently, the stress relaxation and lubrication properties of the hydrogel microsphere system were examined. To verify the stress relaxation performance, the stress curve of the dual‐network hydrogel system was continuously monitored under a 15% fixed strain. The results showed that with the disintegration of the weak cross‐linked disulfide bonds under stress, the stress magnitude of the dual‐network hydrogel system gradually decreased, showing a good stress relaxation effect (Figure 1J). Meanwhile, the lubrication effect of the hydrogel microsphere system was verified by reciprocating friction experiments. We set up the HAMA group injected with the HAMA solution of uncross‐linked liquid as the lubricating fluid, the normal microspheres group adding normal microspheres (prepared using HAMA without stress‐responsive charge reversal functionality) within the HAMA group, and the stress‐responsive microspheres group adding stress‐responsive microspheres within the HAMA group. As shown in Figure 1K, compared with the HAMA group with the friction coefficient (COF) value of 0.15, the COF values of the normal microsphere group (COF = 0.10) and stress‐responsive microsphere group (COF = 0.11) significantly decreased under 10 N mechanical pressure, indicating a certain lubricating performance of the hydrogel microsphere system owing to the bearing lubrication effect.

### 2.3 Abnormal TGF‐β1 Synthesis and Activation Induced by Stress Overload

**FIGURE 2:**
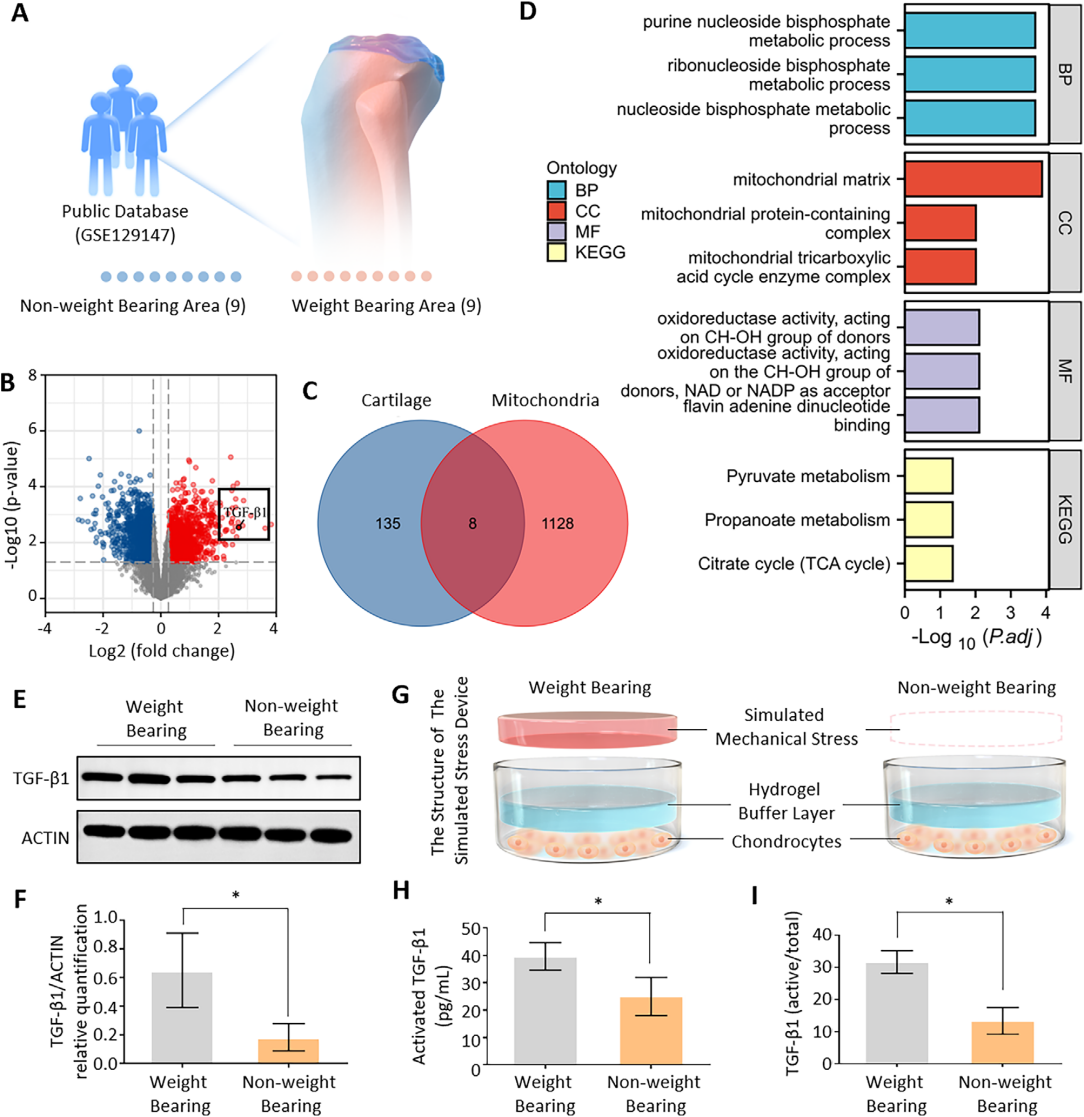
Stress overload induces abnormal TGF‐β1 synthesis and activation and mitochondrial dysfunction. (A) Cartilage tissue sequencing data obtained from a public database (GSE129147). (B) Differentially expressed genes in the cartilage tissue from weight‐bearing and non‐weight‐bearing regions (TGF‐β1 positions are marked with black boxes). (C) Intersection analysis of differentially expressed and mitochondria‐related genes. (D) Functional cluster analysis of eight mitochondrial differentially expressed genes. (E) Rat experiments verified that TGF‐β1 was highly expressed in the stress‐overloaded region (n = 3). (F) Quantitative analysis of western blot results. (G) Schematic representation of cellular experiments verifying that stress overload induces an increase in active TGF‐β1 content. (H) ELISA results of active TGF‐β1 content in different experimental groups (n = 3). (I) ELISA results of the proportion of active TGF‐β1 in different groups (n = 3). (* *p* < 0.05) ELISA, enzyme‐linked immunosorbent assay; TGF‐β1, transforming growth factor beta‐1.

To evaluate the clinical value of the stress‐responsive charge‐reversal hydrogel microsphere system, it is essential to investigate the pathological changes in cartilage tissue under stress‐overload and non‐stress‐overload conditions. To assess the abnormal synthesis and activation of TGF‐β1 in cartilage tissue under stress overload, sequencing data from cartilage samples of weight‐bearing and non‐weight‐bearing areas were obtained from a public database (dataset number: GSE129147). This dataset included 18 cartilage samples, comprising 9 from weight‐bearing areas and 9 from non‐weight‐bearing areas (Figure 2A). Bioinformatics analysis of the sequencing data revealed significantly higher TGF‐β1 expression in cartilage from the weight‐bearing regions compared to that from the non‐weight‐bearing regions (Figure 2B). Furthermore, an overlap of differentially expressed genes with mitochondria‐related gene sets in the cartilage of the weight‐bearing region identified 8 out of 146 differentially expressed genes associated with mitochondria (Figure 2C). Subsequent functional cluster analysis of these eight genes demonstrated their involvement in various mitochondrial physiological activities, such as mitochondrial respiratory chain function and nucleoside diphosphate metabolism (Figure 2D). These results indicate that stress overload significantly affects the mitochondrial function of chondrocytes.

The results of the bioinformatics analysis were subsequently validated through animal experiments in rats, wherein TGF‐β1 expression levels in the cartilage tissues of the weight‐bearing and non‐weight‐bearing regions of the OA model rats were measured using western blotting (Figure 2E). Quantitative analysis of the western blot results showed significantly higher TGF‐β1 expression in the weight‐bearing cartilage compared to the non‐weight‐bearing cartilage (Figure 2F), confirming the findings from the bioinformatics analysis. To further verify the correlation between stress and TGF‐β1 activation, cell experiments were conducted (Figure 2G). The levels of active TGF‐β1 and total TGF‐β1 were quantified using enzyme‐linked immunosorbent assay (ELISA) kits. The results showed that both the content of active TGF‐β1 (Figure 2H) and the ratio of active TGF‐β1 to total TGF‐β1 (Figure 2I) were significantly higher in the cartilage from the weight‐bearing area (40.10 ± 5.36 pg/mL, 32.11% ± 3.81%) compared to that from the non‐weight‐bearing area (25.29 ± 7.07 pg/mL, 13.72% ± 3.99%). In summary, stress overload led to an increase in TGF‐β1 synthesis and the proportion of active TGF‐β1 in chondrocytes, which significantly impacted mitochondrial function in these cells. Finally, we dynamically monitored the activation status of TGF‐β1. We found that within 30 min, as time progressed, the activation rate of TGF‐β1 gradually increased, which further confirmed our conclusion (Figure S10, Supporting Information).

### 2.4 The Hydrogel Microsphere System Alleviates the Dysfunction of Chondrocytes Induced by Abnormal TGF‐β1

**FIGURE 3:**
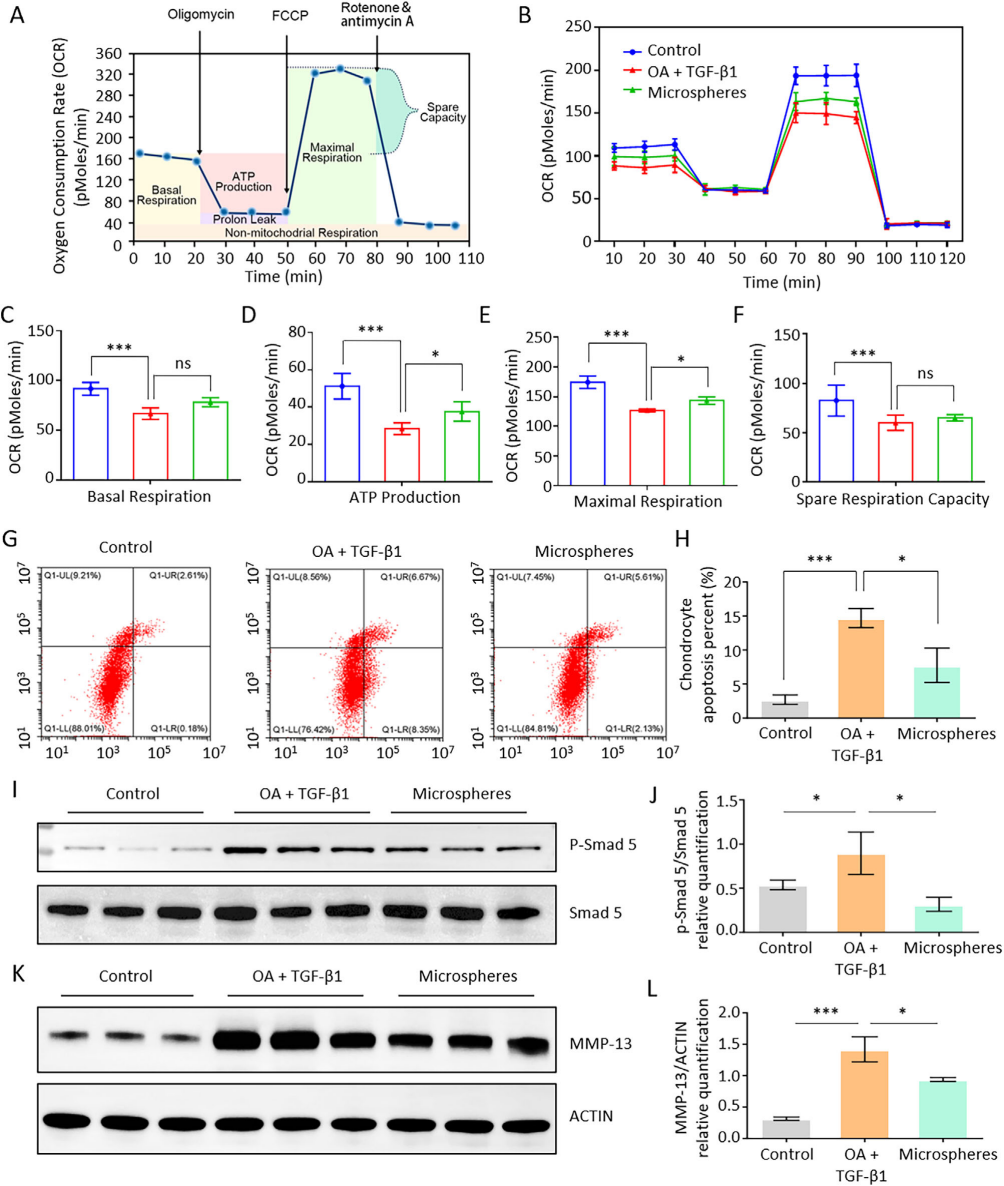
The hydrogel microsphere system alleviates TGF‐β1‐induced dysfunction of chondrocytes. (A) Oxygen consumption rate of chondrocytes in different groups detected using a Seahorse energy metabolism analyzer to evaluate mitochondrial respiratory chain function. (B) Mitochondrial respiratory chain function curves of chondrocytes in different experimental groups (n = 3). (C) Quantitative analysis of basal respiratory function. (D) Quantitative analysis of ATP capacity. (E) Quantitative analysis of maximal respiratory function. (F) Quantitative analysis of reserve respiratory function. (G) Flow cytometry of chondrocytes in different experimental groups. (H) Quantification of apoptosis results of chondrocytes using flow cytometry (n = 3). (I) Detection of Smad 5, p‐Smad 5, and MMP‐13 expression in chondrocytes in different experimental groups. (J) Quantitative analysis of western blot results (n = 3). (ns, not significant, * *p* < 0.05, ** *p* < 0.01, *** *p* < 0.001).

To verify the negative effects of abnormal TGF‐β1 synthesis and activation on chondrocytes, mitochondrial respiratory chain function, cell apoptosis, and activation of related signaling pathways were examined in different chondrocyte microenvironments. First, based on the analysis results from the public database, we believe that stress overload will affect the mitochondrial function of chondrocytes. Additionally, in order to verify the accuracy of this analysis result, we measured the expression levels of the two key genes, CDKN2A and DCAKD (Figure S11, Supporting Information). Subsequently, the oxygen consumption rate of chondrocytes was measured using a Seahorse energy metabolism analyzer, which was used to evaluate the respiratory chain function of mitochondria in chondrocytes (Figure 3A). Untreated chondrocytes were set as the control group, chondrocytes exposed to the OA and high TGF‐β1 concentration microenvironment as the OA + TGF‐β1 group, and chondrocytes treated with hydrogel microspheres as the microsphere group. Subsequently, the mitochondrial respiratory chain function curves of chondrocytes in each experimental group were drawn (Figure 3B), and the important indicators reflected by the curves were quantitatively analyzed (Figure 3C–F). The results showed that the abnormal increase in active TGF‐β1 in the OA + TGF‐β1 group led to the dysfunction of the mitochondrial respiratory chain, including ATP production, maximum respiratory function, and other important indicators, which were significantly lower than those in the control group. Moreover, the mitochondrial respiratory chain function of the simulated treatment group co‐cultured with the hydrogel microsphere system was significantly improved, and the important indicators were significantly higher than those in the OA + TGF‐β1 group.

Subsequently, a preliminary test of the efficacy of the hydrogel microsphere system in inhibiting TGF‐β1‐induced chondrocyte apoptosis was performed by flow cytometry (Figure 3G) and quantitative analysis of the flow cytometry result (Figure 3H). The results showed that the apoptosis rate of chondrocytes exposed to excessive TGF‐β1 (OA + TGF‐β1 group) (2.70 ± 0.69%) was significantly higher than that of the control group (14.70 ± 1.41%). The treatment of chondrocytes with hydrogel microspheres loaded with LSKL significantly reduced TGF‐β1‐induced chondrocyte apoptosis (7.74 ± 2.53%). These results indicate that the hydrogel microsphere system can effectively release LSKL drugs to block the effect of TGF‐β1, thereby alleviating chondrocyte apoptosis. In conclusion, our constructed hydrogel microsphere system can effectively inhibit chondrocyte apoptosis induced by the excessive TGF‐β1 synthesis and activation, which is beneficial to the repair and regeneration of cartilage tissue.

Finally, the downstream signaling pathways induced by abnormally synthesized versus activated TGF‐β1 were examined. Similarly, untreated chondrocytes were set as the control group, chondrocytes treated with activated TGF‐β1 in the simulated OA microenvironment as the OA+TGF‐β1 group, and chondrocytes treated with the hydrogel microsphere system were as the microsphere group. The protein levels of Smad 5, p‐Smad 5, and MMP‐13 in different groups of chondrocytes were detected (Figure 3I). Quantitative analysis showed that the phosphorylation of Smad 5 protein in the OA+TGF‐β1 group was significantly higher than that in the control group. However, the phosphorylation of Smad 5 protein in the microsphere group was significantly inhibited, which was significantly lower than that in the OA+TGF‐β1 group. Subsequently, the synthesis of the MMP‐13 protein, which is downstream of the Smad 5 signaling pathway, showed the same pattern. Owing to the excessive activation of the Smad 5 signaling pathway, the synthesis of MMP‐13 in the OA+TGF‐β1 group was significantly higher than that in the control group. In contrast, the microsphere group inhibited the synthesis of MMP‐13 due to the inhibition of the Smad 5 signaling pathway (Figure 3J). Of course, we also set up a blank microsphere group without any drugs. By comparing it with the OA+TGF‐β1 group, we controlled for irrelevant variables (Figure S12, Supporting Information). Meanwhile, in order to lay a foundation for future research, we investigated the activation status of the MARK pathway downstream of TGF‐β1. According to the western blot results, under TGF‐β1 induction, the classical ERK signaling pathway and the P38 signaling pathway were both activated and could be inhibited by microspheres (Figure S13, Supporting Information).

Therefore, our constructed hydrogel microsphere system can effectively inhibit the TGF‐β1‐induced activation of the Smad 5 signaling pathway and the synthesis of the MMP‐13 protein, thereby alleviating extracellular matrix degradation.

### 2.5 The Hydrogel Microsphere System Alleviates the Stress Injury of the Knee Joint

**FIGURE 4:**
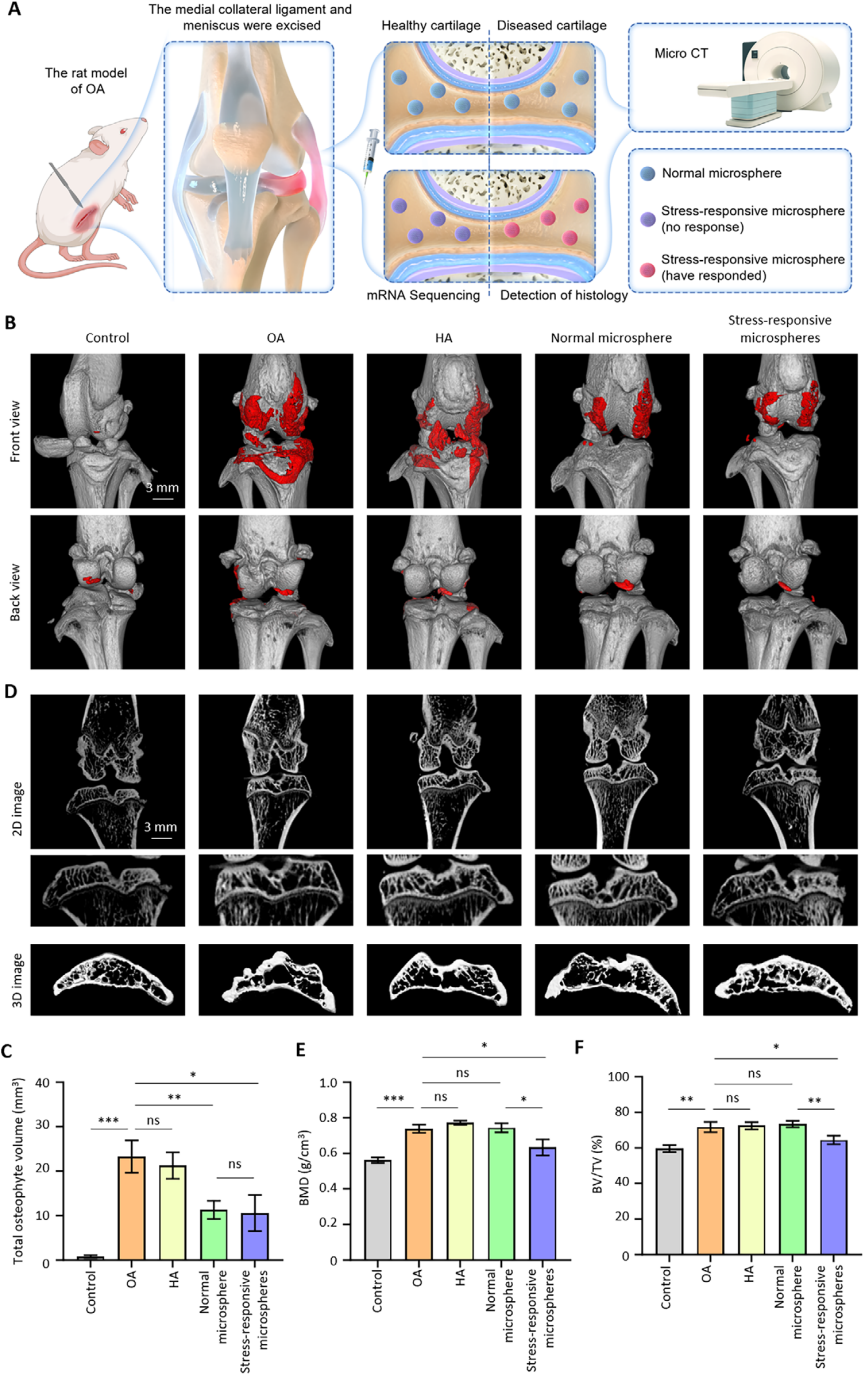
The hydrogel microsphere system alleviates the stress injury of the knee joint. (A) Schematic diagram of the rat OA model construction and animal experiment design. (B) 3D reconstruction of the micro‐CT image of the rat knee joint (the red area is hyperplastic osteophytes). (C) Quantitative analysis of the volume of hyperplastic osteophytes (n = 3). (D) Micro‐CT images of the subchondral bone of the knee joint. (E) Quantitative analysis of bone mineral density (BMD) values (n = 3). (F) Quantitative analysis of the bone fraction volume (BV/TV) (n = 3). (ns, not significant, * *p* < 0.05, ** *p* < 0.01, *** *p* < 0.001).

To verify the efficacy of the hydrogel microsphere system in vivo, a rat OA model was established by surgically removing the medial collateral ligament and medial meniscus. In this model, the lateral side of the knee joint served as the normal stress area, whereas the medial side was the stress‐overloaded area. Firstly, we injected hydrogel microspheres loaded with fluorescently labeled nanoparticles into the joint cavity to verify the retention performance of the hydrogel microsphere system in the joint cavity. The results of in vivo fluorescence imaging showed that the hydrogel microsphere system could effectively remain in the joint cavity for about 3 weeks. This laid a solid foundation for its therapeutic function (Figure S14, Supporting Information). Subsequently, OA rats were divided into several experimental groups for treatment and subsequent testing according to the experimental design (Figure 4A). To validate the efficacy of the hydrogel microsphere system for OA treatment via weak cross‐linking covalent bonding and a bearing lubrication mechanism, micro‐computed tomography (CT) was performed on the knee joints of rats after 12 weeks of treatment. The untreated rats were set as the control group, rats treated with phosphate‐buffered saline (PBS) as the OA group, rats treated with HA injection commonly used in clinical practice as the HA group, rats treated with normal hydrogel microspheres as the normal microsphere group, and rats treated with hydrogel microspheres with stress‐responsive charge‐reversal function as the stress‐responsive microsphere group. Osteophyte proliferation in the knee joints of the rats in each group was detected by the three‐dimensional reconstruction function of the micro‐CT instrument (Figure 4B). Quantitative analysis of osteophyte volume revealed that the osteophyte volume in the OA group was significantly higher than that in the control group, indicating that stress overload and increased friction placed a heavy burden on the knee joint. Moreover, no significant difference was found between the HA and OA groups, indicating that the existing clinical treatment options for OA are not effective. Osteophytes in the normal microsphere and stress‐responsive microsphere groups were significantly smaller than those in the OA group, indicating that the bearing lubrication mechanism of the hydrogel microsphere system can significantly improve the mechanical environment of the knee joint. Thus, osteophyte proliferation was reduced (Figure 4C). Additionally, abnormal remodeling of the subchondral bone is another manifestation of stress overload. Therefore, 2D micro‐CT images and 3D reconstruction images of the subchondral bone were examined (Figure 4D). Furthermore, to quantitatively reflect subchondral bone remodeling, the bone mineral density (BMD) (Figure 4E) and bone fraction volume (BV/TV) (Figure 4F) of the subchondral bone were measured. The results showed that the BMD and BV/TV values in the OA group (73.87 ± 0.02 g/cm^3^, 71.74% ± 2.84%) were significantly higher than those in the control group (56.13 ± 0.02 g/cm^3^, 59.61% ± 2.00%), indicating that stress overload and increased elastic stress led to abnormal subchondral bone remodeling. Moreover, no significant difference in BMD and BV/TV values was noted between the HA, normal microsphere, and OA groups, indicating that HA injection and normal microspheres could not alleviate the damage caused by elastic stress. However, the BMD and BV/TV values in the stress‐responsive microsphere group (63.40 ± 0.04 g/cm^3^, 64.49% ± 2.41%) were significantly lower than those in the OA group. At the same time, we also conducted quantitative analyses on the bone trabecular thickness (Tb.Th) and the trabecular separation (Tb.Sp) in the subchondral bone. The results were consistent with the above analysis. The stress‐responsive microspheres were able to effectively alleviate the hyperplasia and remodeling of the trabeculae in the subchondral bone (Figure S15, Supporting Information). These results indicate that the constructed stress‐responsive charge‐reversal hydrogel microspheres can absorb the excess energy induced by elastic stress through the breakage of weakly cross‐linked covalent bonds, thus protecting the subchondral bone.

In summary, the developed stress‐responsive charge‐reversal hydrogel system effectively alleviates osteochondral hyperplasia and abnormal subchondral bone remodeling in the knee joint under stress overload. It achieves this by utilizing weakly cross‐linked covalent bonds and a bearing lubrication mechanism, thereby creating an optimal environment for cartilage repair and regeneration.

### 2.6 Effect of Hydrogel Microspheres Matching TGF‐β1 Activation in the Knee Joint

The key to the matching TGF‐β1 activation and treatment of OA is whether the hydrogel microsphere system can accurately identify the stress‐overload area. Therefore, the constructed stress‐responsive charge‐reversal hydrogel microspheres were verified whether they could identify the stress‐overloaded region to reveal their advantages over the normal hydrogel microspheres.

**FIGURE 5:**
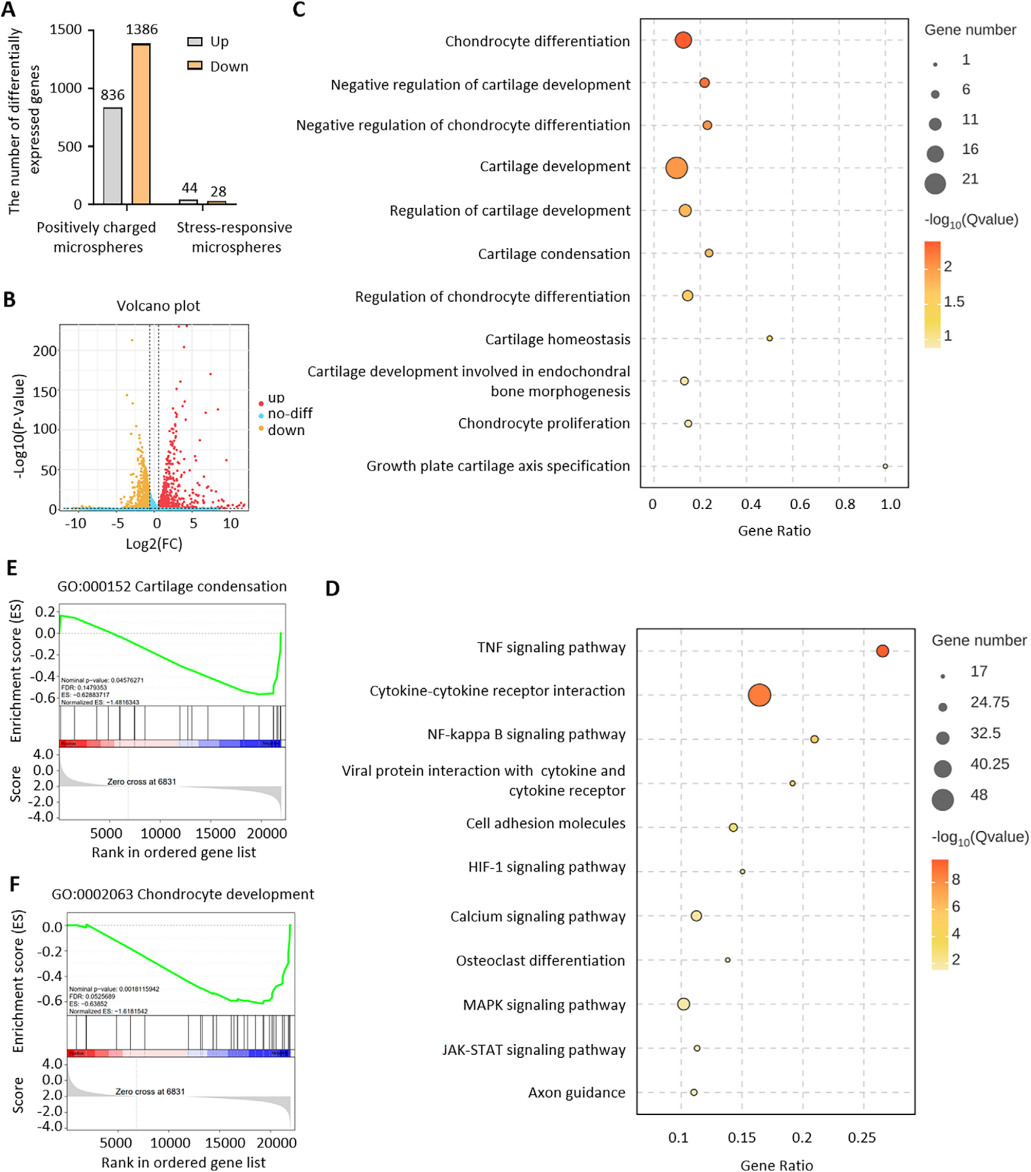
Protection of healthy cartilage via match the activation of TGF‐β1. (A) Effects of different hydrogel microsphere designs on healthy cartilage. (B) Differentially expressed genes in healthy cartilage under the influence of positively charged hydrogel microspheres. (C) Cartilage health‐related GO items. (D) Cartilage health‐related KEGG items. (E) GSEA enrichment score of cartilage condensation item. (F) GSEA enrichment score for chondrocyte development.

First, the recognition effect of the stress‐responsive charge‐reversal hydrogel microsphere system on healthy cartilage was demonstrated. Positively charged hydrogel microspheres and stress‐responsive charge‐reversal hydrogel microspheres were injected into the knee joint of OA rats, and the lateral cartilage of the knee joint (healthy cartilage) was sampled for sequencing. The sequencing results showed that the cartilage of the positively charged microsphere group was affected by excessive LSKL, and 836 differentially upregulated genes and 1386 differentially downregulated genes were identified. However, only 44 differentially upregulated and 28 differentially downregulated genes were identified in the stress‐responsive microsphere group, indicating that the stress‐responsive charge‐reversal hydrogel microsphere system effectively identified the healthy cartilage area, accurately controlled the drug release, and thus protected the healthy cartilage (Figure 5A).

Furthermore, to demonstrate the advantages of the stress‐responsive charge‐reversal hydrogel microsphere system and clarify the side effects of non‐selective targeting and treatment, the differentially expressed genes in the positively charged microsphere group were further analyzed (Figure 5B). Among them, the Gene Ontology (GO) items with the highest enrichment scores were mainly concentrated in the molecular function and biological process categories (Figure S16A, Supporting Information). The Kyoto Encyclopedia of Genes and Genomes (KEGG) items with the highest enrichment scores were mainly concentrated on environmental information processing, human disease, and organismal systems (Figure S16B, Supporting Information). Subsequently, GO items related to cartilage health were screened, and the results showed that the enrichment of differentially expressed genes in 11 GO items was significant (*p* < 0.05). These included the negative regulation of cartilage development, negative regulation of chondrocyte differentiation, and cartilage development involved in endochondral bone morphogenesis (Figure 5C). These results indicate that the indiscriminate inhibition of TGF‐β1 can have significant adverse effects on healthy cartilage. KEGG items related to cartilage health were also screened, and KEGG items with the top 11 enrichment scores were shown (*p* < 0.05), including the tumor necrosis factor signaling pathway, cytokine–cytokine receptor interaction, and NF‐kappa B signaling pathway. Excessive activation of these pathways may negatively affect healthy soft cartilage (Figure 5D). Furthermore, in order to validate our KEGG analysis results, we used western blot technology to verify the activity of the TNF signaling pathway and the NF‐κB signaling pathway. The experimental results show that the excessive release of positively charged nanoparticles does indeed activate the TNF signaling pathway and NF‐κB signaling pathway (Figure S17, Supporting Information).

Finally, GSEA enrichment analysis of differentially expressed genes was performed, and the results showed that in cartilage‐related items, the enrichments of GO:000152 cartilage condensation (Figure 5E) and GO:0002063 chondrocyte development (Figure 5F) were significant (*p* < 0.05), and the enrichment score was negative. This finding indicated the downregulation of the overall expression of the gene set in this item. Thus, the negative effects of non‐selective targeting and treatment on healthy cartilage are further confirmed.

### 2.7 Stress‐Responsive Charge‐Reversal Hydrogel Microsphere System for OA Treatment

**FIGURE 6:**
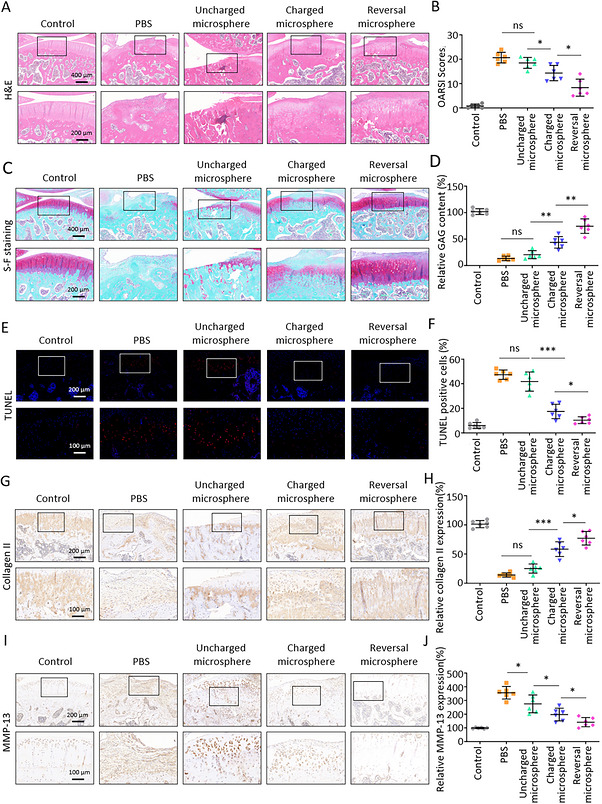
Efficacy of stress‐responsive charge‐reversal hydrogel microspheres for OA Treatment. (A) Representative images of H&E staining. (B) OARSI scores of the articular cartilage in each group. (C) Representative images of Safranin O‐fast green staining. (D) Relative glycosaminoglycan (GAG) content in each group. (E) Representative sections showing TUNEL staining for apoptotic cells. (F) Quantification of TUNEL‐positive cells. (G) Representative sections showing collagen II staining for chondrocytes. (H) Relative collagen II expression in each group. (I) Representative sections showing MMP‐13 staining of chondrocytes. (J) Relative MMP‐13 expression in each group. (ns, not significant, *p < 0.05, **p < 0.01, *** *p* < 0.001). OARSI, Osteoarthritis Research Society International.

To verify the efficacy of the treatment for diseased cartilage using the stress‐responsive charge‐reversal hydrogel microsphere system, the medial knee joints of OA rats were examined after group treatment (Figure S18, Supporting Information). Rats in PBS group were injected with PBS buffer after operation, and Uncharged microsphere group were injected with uncharged hydrogel microspheres (the same as the aforementioned normal microspheres), and Charged microsphere group were injected with positively charged hydrogel microspheres, and the Reversal microsphere group was injected with stress‐responsive charge‐reversal hydrogel microspheres. The knee joints were harvested, decalcified, and sectioned for paraffin embedding, followed by morphological and immunofluorescence staining. The results of hematoxylin and eosin (H&E) staining (Figure 6A) revealed that the tissue structure in the control group was intact, with uniform staining and no noticeable lesions or damage. The PBS group and uncharged microsphere group, however, exhibited poor staining, with loose tissue, uneven staining, and areas showing lesions or damage. The charged microsphere group showed moderate staining, with relatively preserved tissue structure and more uniform staining, although some areas still showed lesions or damage. Conversely, the reversal microsphere group displayed excellent staining, complete tissue structure, and uniform staining, with a significant reduction in diseased or damaged areas. Quantitative analysis using the Osteoarthritis Research Society International (OARSI) scoring system (Figure 6B) confirmed these findings. The PBS group and uncharged microsphere group showed the worst results, whereas the charged microsphere group exhibited moderate improvements. The reversal microsphere group performed significantly better than the others, demonstrating the potential of the stress‐responsive charge‐reversal hydrogel microsphere systems in the treatment of OA.

Subsequently, Safranin O‐fast green staining was performed on the knee samples to assess the effects of different treatments on cartilage health (Figure 6C). In the PBS group and uncharged microsphere group, the cartilage tissue showed marked signs of degeneration. The charged microsphere group exhibited moderate protection of the cartilage tissue, though some areas still showed signs of degeneration. Conversely, the reversal microsphere group demonstrated the best protective effect, with relatively intact cartilage structure and a significant reduction in degeneration. The treatment effect of each group was further assessed through quantitative analysis of glycosaminoglycan (GAG) content (Figure 6D). The results showed that the GAG content in the PBS group and uncharged microsphere group was the lowest, confirming its lack of protective effect on the cartilage tissue. The GAG content in the charged microsphere group was significantly higher than that in the uncharged microsphere group, indicating that the charged microspheres helped mitigate cartilage matrix degradation to some extent. The GAG content in the reversal microsphere group was significantly higher than that in the charged microsphere, demonstrating that the stress‐responsive charge‐reversal microspheres had the most significant protective effect on the cartilage tissue.

Then, TUNEL staining was performed to detect chondrocyte apoptosis (Figure 6E), and positively stained cells (green cells) were quantified (Figure 6F). The results showed marked effects of different treatments on chondrocyte apoptosis. The apoptosis rate of chondrocytes in the PBS group and uncharged microsphere group was the highest, and 47.39% ± 3.79% and 41.78% ± 7.82% of the cells were positively stained. The apoptosis rate of the charged microsphere group was moderate, 17.45% ± 5.83% of the cells were positively stained, and apoptosis still existed in some areas. The apoptosis rate of the Reversal microsphere group was the lowest, and 10.33% ± 2.80% of the cells were positively stained, respectively. The cartilage structure was relatively intact, and apoptosis was significantly reduced. These results signify that the stress‐responsive charge‐reversal microspheres have a clear advantage in protecting chondrocytes.

Collagen II is an important biomarker for assessing cartilage health. Subsequently, the expression of collagen II was examined by immunohistochemistry in the five experimental groups (Figure 6G), and positively stained cells were quantified (Figure 6H). Specifically, the expression of collagen II in the PBS group and uncharged microsphere group was at a low level, and the proportions of collagen II‐positive cells were 14.10% ± 3.55% and 25.16% ± 7.79%, which indicated severe damage to the cartilage tissue. The expression of collagen II in the charged microsphere group was moderate, and the proportion of collagen II‐positive cells was 58.45% ± 12.64%. Despite the high expression in some areas, the overall condition was improved. The reversal microsphere group had the highest expression of collagen II, and the proportion of collagen II‐positive cells were 77.16% ± 11.63%, respectively. The cartilage structure was relatively intact, and the damage was significantly reduced, showing a better cartilage‐protection effect.

Matrix metalloproteinase‐13 (MMP‐13) was nearly absent in the healthy cartilage but significantly increased in diseased cartilage. To assess the effect of different treatments on MMP‐13 expression, five experimental groups were stained by immunohistochemistry (Figure 6I), and quantitative analysis was performed (Figure 6J). The results showed a very low expression level of MMP‐13 in the control group, and the cartilage tissue remained healthy. The cartilage tissue of the PBS group was severely damaged, and the expression of MMP‐13 was significantly increased (356.7% ± 45.91%). Some regions in the uncharged microsphere group still had high expression; however, it was lower than that of the PBS group. The expression levels of MMP‐13 in the charged microsphere group (197.2% ± 46.17%) and Reversal microsphere group (141.7% ± 33.79%) were the lowest, showing the best cartilage‐protection effect.

## 3 Conclusion

In the OA pathological microenvironment, the distribution of mechanical stress varies spatially. Stress overload can stimulate abnormal TGF‐β1 synthesis and activation in cartilage tissue, leading to chondrocyte apoptosis and extracellular matrix degradation. However, spatial heterogeneity in the distribution of TGF‐β1 exists within cartilage tissue due to the correlation between abnormal TGF‐β1 synthesis and stress levels. If TGF‐β1 is suppressed indiscriminately, healthy cartilage will be negatively affected. With the development of biomaterial science, it has brought about possibilities for solving the aforementioned problems [19, 20, 21]. Herein, a stress‐responsive charge‐reversal hydrogel microsphere system was developed for matching TGF‐β1 activation and treatment of OA. The stress‐responsive breakage of weakly cross‐linked covalent bonds in the hydrogel microsphere system was used to accurately identify the magnitude of stress, while the interaction between cleavage of weakly cross‐linked disulfide bonds under stress and charge‐reversal messenger molecules reversed the charge of nanoparticles in response to mechanical stress. The hydrogel microsphere system can lubricate and alleviate stress in the articular cavity, and the matching between stress‐induced charge reversal and spatiotemporal distribution differences in the pathological microenvironment of cartilage enables the selective release of reversed positively charged nanoparticles. These nanoparticles can penetrate deeply into the cartilage matrix and match the activation of TGF‐β1. This was made possible by the identification and matching of stress‐induced differences in spatial distribution and the pathological microenvironment. This study provides a new idea for the development of biomaterial regulatory systems that match the pathological microenvironment.

The hydrogel system we have constructed has considerable application value in other fields as well. For example, other mechanically stressed tissues: this includes tendinopathy, intervertebral disc degeneration, and ligament injuries, where localized mechanical overload contributes to inflammation and tissue breakdown. Wound healing and fibrosis: in skin or internal organs subjected to abnormal mechanical tension (e.g., hypertrophic scars, and liver fibrosis), the system could deliver anti‐fibrotic agents specifically to stress‐activated myofibroblasts. Cardiovascular diseases: in atherosclerotic plaques or hypertensive vessels, regions of high shear or tensile stress could trigger localized drug release to stabilize vulnerable lesions. Cancer microenvironments: solid tumors often exhibit elevated interstitial pressure and matrix stiffness; this system could be tuned to respond to mechanical cues in the tumor stroma for targeted chemotherapy or immunomodulation.

This study also has some limitations and requires further exploration. For instance, the relationship between mechanical stress and the downstream signaling pathways of TGF‐β1 requires further exploration, which will lay a solid foundation for the expanded application of this hydrogel microsphere system. Meanwhile, in future research, large animal experiments (such as on dogs) and human experiments are necessary. This will provide practical evidence for the clinical application of the hydrogel microsphere system. In conclusion, this hydrogel microsphere system possesses excellent scalability and clinical translational value.

## 4 Materials and Methods

### 4.1 Synthesis of the Polymeric Materials

I) HAMA: The generation method of HAMA was similar to that described previously [22]. Briefly, 1000 mg of hyaluronic acid (HA) was weighed and dissolved in 50 mL of deionized water. Next, 2 mL of methacrylate amide (MA) was added dropwise to the solution and stirred well. Then, 2 mL of 5 M NaOH was added dropwise. The mixture was incubated overnight. After centrifugation, the supernatant was dialyzed for 3 days, frozen, and lyophilized. II) HASH: Overall, 500 mg of HA was dissolved in 150 mL of deionized water, and 255 mg of HOBt (1.88 mmol) was added, followed by 450 mg of mercaptoethylamine (1.88 mmol). Next, 115 mg EDC (0.6 mmol) was added, and the reaction was incubated for 24 h. Then, 385.5 mg of DTT (2.5 mmol) was added and incubated for 24 h. The mixture was dialyzed for 3 days, frozen, and lyophilized. III) DSPE‐PEG‐Nap‐N_3_: Overall, 46.83 g of DSPE‐PEG‐COOH (58.53 mmol), 214 mg of catalyst DMAP (1.75 mmol), and 1.45 g of dehydrating agent DCC (7.03 mmol) were added to a 250 mL Schlenk flask, which was then filled with argon. Next, 100 mL of anhydrous THF was added, and the mixture was stirred on ice. Then, 2.12 g of Nap‐N_3_ (8.18 mmol) was dissolved in 10 mL of anhydrous THF, and the solution was slowly added to the above mixture. After a 4‐h incubation on ice, the reaction was continued for 48 h at room temperature. After purification, the product was dialyzed for 3 days and freeze‐dried to obtain a viscous solid. The schematic diagram of the above polymer synthesis was shown in Figure S19.

### 4.2 Synthesis of Charge‐Reversal Liposomes

Chloroform (1.5 mL) was used to dissolve 12 mg of the phospholipid layer reagent (lecithin: cholesterol: DSPE‐PEG‐Nap‐N_3_ = 4:1:1). *N*‐ (benzoyl thio) benzamide (0.2 mg) was added as a messenger molecule to a silane‐based flask. Rotary evaporation was used to remove the organic solvent, obtaining a hyaline lipid film. The LSKL (a tetrapeptide composed of leucine, serine, lysine, and leucine) solution (10 mg/mL, 17.5 mM) was prepared in PBS (pH = 7.4), added to a pear‐shaped flask, hydrated at 37°C to form the phospholipid membrane, and sonicated using an ultrasonic probe (20%, 10 min) to obtain charge‐reversal functional nanoliposomes. The schematic diagram of liposome synthesis is shown in Figure S20.

### 4.3 Characterization of the Biological Materials

I) Morphological observation: The morphology of the microspheres (Tecnai G2, Spirit Biotwin) was observed using scanning electron microscopy. The morphology of the nanoliposomes was observed using transmission electron microscopy (Sirion 200, FEI). II) Element detection: The hydrogel microspheres were analyzed for various elements using the energy spectrum mode of scanning electron microscopy. III) Particle size and potential: The particle size and potential of the nanoparticles were examined using a DLS (Zetasizer Nano S, Malvern). IV) Mechanical property testing: The stress–strain curve of the hydrogel system was measured using a universal mechanical tester (68TM, Instron).

### 4.4 Stress Relaxation Property Testing

The dual‐network hydrogel system was compressed to a 15% fixed strain with a deformation rate of 1 mm/min using a universal mechanical tester (68TM, Instron). The 15% strain ratio was maintained while recording the curves of the detected stresses over time.

### 4.5 Bearing Lubrication Performance Testing

A tribological test was carried out using a friction and abrasion tester (UMT‐3, Bruker Nano Inc., Germany). Using the same experimental conditions, the lubrication properties of the hydrogel microsphere system suspended in PBS solution and simulated synovial fluid were evaluated and compared with those of the control group without microspheres. The friction cycle test was performed at room temperature for 600 s. The wear of the lower surface was observed under a light‐field microscope, and the friction coefficient was determined and analyzed.

### 4.6 Western Blot Analysis

The steps for Western blot were as described previously [23]. Briefly, the cultured cells were washed twice with pre‐chilled PBS, RIPA buffer was added, and the cells were lysed for 30 min on ice. The samples were centrifuged at 12,000 rpm for 20 min at 4°C, and the supernatants were collected. The protein concentration was determined using the BCA protein quantification kit (Thermo Scientific, USA). A 10% SDS‐PAGE separating gel and a 5% stacking gel were prepared. The protein samples were mixed with 5× loading buffer and heated at 95°C for 5 min. After loading, the gel was initially run at 80 V followed by 120 V through the separation gel. The proteins were transferred to a PVDF membrane at 100 V for 90 min and blocked in 5% skim milk in TBST buffer for 1 h at room temperature. The membrane was then incubated overnight at 4°C in diluted primary antibody (Smad 5: 12167‐1‐AP, Proteintech; p‐Smad 5: bsm‐52206R, Bioss; MMP‐13: 18165‐1‐AP, Proteintech) followed by three washes with TBST for 10 min each. The membranes were incubated in diluted secondary antibody for 1 h at room temperature, and the membranes were washed three times with TBST for 10 min each. Chemiluminescence was performed using the ECL color development kit. The resulting images were captured, and ImageJ software (version: 2.14.0/1.54f) was used to analyze the gray value of the bands and to calculate the relative amount of the target proteins.

### 4.7 Weight Bearing Experiment

Chondrocytes were first seeded into 6‐well plates at a density of 1 × 10^4^ cells per well. Initially, culture was performed for 8 h under standard conditions; then, the medium was changed, and culture was performed for an additional 16 h. Subsequently, a 4 wt% HAMA hydrogel layer (cured under UV light) was applied on the top of the chondrocytes to mimic the extracellular matrix. To simulate mechanical stress similar to that experienced by chondrocytes in joint environments, a 100‐g experimental weight (Nanjing Su Measurement and Measuring Instrument Co. Ltd., China) was placed on the top of the hydrogel layer, and through calculation, the pressure exerted on the chondrocytes is approximately 150 kPa. Subsequently, at time points of 0, 10, 20, and 30 min, we extracted the cell supernatants to dynamically observe the activation status of TGF‐β1.

### 4.8 ELISA

The concentration of active TGF‐β1 was measured in the conditioned media using an ELISA development kit (R&D Systems) based on the manufacturer's instructions. As instructed by the vendor‐provided manual, the active TGF‐β1 was measured directly from the conditioned media while the total TGF‐β1 was measured after treating with acid.

### 4.9 Mitochondrial Respiratory Chain Assay

Calibration was done according to the Seahorse XF analyzer (Agilent Technologies, XFe24) manual, and oligomycin (stored at 2.5 mmol/L, used at 1 µmol/L), FCCP (stored at 2.5 mmol/L, used at 1 µmol/L), FCCP (stored at 2.5 mmol/L, used at 1 µmol/L), and antimycin A (2.5 mmol/L stored, 1 µmol/L used) were prepared. A total of 100 µL of a single cell suspension (10^5^ cells/mL) from each group was incubated at 37°C until the cells adhered. Next, 150 µL of medium was added, followed by drugs, and incubated for 72 h before detection. Then, 175 µL of the original medium was discarded, the cells were rinsed twice with 600 µL of hippocampal test medium, and 450–525 µL of medium was added to observe cell continuity under a microscope. The plates were incubated in a CO_2_‐free incubator for 1 h. Based on the experimental design, 75 µL of the drug was added to each of the four dosing tanks per well. The instrument was calibrated, and the cell plate was analyzed. At the end of the assay, the cells in the sample wells were collected, centrifuged at 1000 rpm for 5 min, and stored at −80°C.

### 4.10 Apoptosis Detection

The cells were cultured to a logarithmic growth phase at 37°C in 5% CO_2_ in the appropriate medium and treated with various drug concentrations. Each group contained three duplicate samples and was incubated for 24 h. The cells were washed twice with pre‐chilled PBS, incubated with EDTA‐free trypsin, and centrifuged at 300 g for 5 min. The cells were resuspended in 100 µL of 1× binding buffer from the Annexin V‐FITC Apoptosis detection kit (Beyotime, C1062S, China). Next, 5 µL of FITC‐Annexin V and 5 µL propidium iodide (PI) were added, mixed gently, and incubated at room temperature in the dark for 10–15 min. The cells were resuspended in 400 µL of 1× binding buffer and analyzed by flow cytometry (FACSCalibur, USA). Excitation of FITC‐Annexin V was done at 488 nm, and fluorescence was detected at 530 nm. Nuclear staining was performed using PI, and the fluorescence was detected at 617 nm. Flow cytometry software was used to record the fluorescence intensity data, and FlowJo software (version: 10.8.1) was used to analyze the data and calculate the proportion of early apoptosis, late apoptosis, and necrotic cells.

### 4.11 Live and Dead Cell Staining

Cells were seeded in six‐well plates and cultured in the appropriate medium at 37°C in a 5% CO_2_ atmosphere until they were in a logarithmic growth phase. Based on the experimental design, different materials were added to the co‐culture, and each group was treated for 48 h in triplicate. After treatment, the cells were washed twice with PBS to remove residual medium and serum. A working solution was prepared based on the instructions in the live/dead cell staining kit (Beyotime, C2015S, China). Briefly, 1 µL of 1000× PI and 1 µL of 1000× calcein AM were added to 1 mL of assay buffer and mixed. The washed cells were mixed with the staining working solution, followed by 1 mL of the calcein AM/PI assay working solution. After incubating at 37°C in the dark for 30 min, the cells were observed by fluorescence microscopy. Calcein AM showed green fluorescence (Ex/Em = 494/517 nm), and PI exhibited red fluorescence (Ex/Em = 535/617 nm). The entire process was performed in the dark. Live cells showed green fluorescence, and dead cells exhibited red fluorescence.

### 4.12 Animal Model Construction

Eight‐week‐old SD rats weighing 220 ± 20 g were randomly divided into experimental and control groups, with six rats in each group. The rats were maintained in standard laboratory animal housing. They were anesthetized with Zoleti‐50 (10 mg/kg) under general anesthesia. The medial articular hair of the hind limbs was shaved, and the surgical area was sterilized with iodophor or 75% alcohol. The skin and fascia were incised, the knee was exposed, and the medial meniscus and medial collateral ligament were removed. The muscle and skin were sutured with absorbable sutures and re‐sterilized at the end of surgery. The rats were placed in a warm environment and observed to ensure full recovery before returning to the rearing cages. Appropriate antibiotics were administered to prevent infection for 3 consecutive days after surgery. The activity of the rats was observed within 1 week after the operation. Reduced autonomous activity and limited mobility were recorded.

### 4.13 MRNA Sequencing

The harvested tissues were rinsed with saline to remove blood stains and dirt. After excluding non‐study tissues, the tissues were blotted dry, cut into small pieces, and placed into 1.5 mL tubes. RNA was extracted using TRIzol. Chloroform was added, and the mixture was shaken for 15 s, allowed to stand for 2–3 min, and centrifuged at 12,000 rpm for 15 min at 4°C. The supernatant was transferred to a new tube. An equal volume of isopropanol was added, mixed, and allowed to stand for 10 min. After centrifugation at 12,000 rpm at 4°C for 10 min, the supernatant was discarded. The RNA precipitate was washed twice with 75% ethanol, air‐dried, and dissolved in RNase‐free water. The mRNA was enriched with Oligo(dT) magnetic beads, and first‐strand cDNA was synthesized using M‐MuLV reverse transcriptase with random oligonucleotide primers and fragmented mRNA as the template. The RNA strands were degraded via RNase H, and second‐strand cDNA was synthesized using the DNA polymerase I system. Double‐stranded cDNA was purified, end‐repaired, A‐tailed, and ligated to A sequencing adaptors. The cDNA fragments of approximately 200 bp were purified using AMPure XP beads and subjected to PCR amplification. The PCR products were purified to obtain a library. RNA concentration was quantified using a Qubit 2.0 Fluorometer, and RNA integrity was assessed using an Agilent 2100 Bioanalyzer.

### 4.14 Micro‐CT Detection

The Micro‐CT was used as described previously [24]. Briefly, the rat knee joints were removed, rinsed with normal saline, dried, and fixed in 10% formalin solution for 24 h. After fixation, the plates were rinsed with PBS buffer to remove any residual fixative and dried at room temperature. The Micro‐CT device was preheated and calibrated, and the scanning parameters were set. A filtering algorithm was applied to remove image noise and improve the image resolution. The obtained multiangle X‐ray images were reconstructed into 3D images using a reconstruction algorithm.

### 4.15 Histology and H&E Staining

After sampling the rat knee joint, the specimens were fixed in 10% formalin solution for 24 h, followed by dehydration and paraffin embedding. The embedded tissue blocks were cut into 5–10‐µm thick sections using a microtome. The sections were then deparaffinized and hydrated sequentially in xylene and a graded series of ethanol concentrations. The sections were stained with hematoxylin solution for 5–10 min, and excess dye was removed. Immerse sections in 1% hydrochloric ethanol for a few seconds and place into a weak alkaline solution (e.g., ammonia or soda) for several minutes to restore the blue color of nuclei. The sections were stained with eosin staining solution for 1–3 min, rinsed with tap water, dehydrated sequentially in a graded ethanol series (70%, 85%, 95%, and 100%), and treated with xylene. The sections were mounted with neutral gum, covered with a cover slip, dried, and analyzed via microscopy.

### 4.16 TUNEL Staining

The tissue sections were fixed overnight in 4% paraformaldehyde, deparaffinized in xylene, and sequentially rehydrated in a graded series of ethanol solutions and distilled water. The sections were incubated with proteinase K for 30 min to increase permeability, then incubated with a blocking solution for 10 min to prevent non‐specific binding. Some sections were treated with DNase I as a positive control. The sections were incubated with the TUNEL reaction mixture for 1 h at 37°C, followed by incubation with a streptavidin–HRP conjugate for 30 min. The color was developed with DAB substrate solution. The slides were then rinsed with PBS, counterstained with hematoxylin for 2–5 min, dehydrated, and mounted.

### 4.17 Immunohistochemical Staining

Tissue samples were obtained from the rat knee joints, fixed, dehydrated, embedded, and sectioned. The sections were deparaffinized in xylene, rehydrated sequentially in 100%, 95%, 85%, and 70% ethanol, and rinsed with distilled water. Antigen retrieval was performed by microwaving in citrate buffer for 10 min. Endogenous peroxidase activity was blocked by incubating the sections with 3% hydrogen peroxide for 10 min, and non‐specific binding was blocked with 3% BSA for 1 h. The sections were incubated overnight at 4°C with primary antibodies to Collagen II and MMP‐13 (ServiceBio, China), washed, and incubated for 1 h with HRP‐labeled secondary antibodies (ServiceBio, China). The color was developed with DAB substrate solution, followed by rinsing with distilled water and counterstaining with hematoxylin for 2–5 min. The sections were dehydrated and mounted.

### 4.18 Statistical Analysis

A two‐tailed analysis of variance (ANOVA) and a t‐test were used to analyze the significant differences between the two groups of samples. Comparisons involving more than two groups were done using a one‐way ANOVA with a post hoc test. All experiments included at least three groups of parallel, repeated samples. Statistical significance was set at *p* < 0.05 and denoted as *p < 0.05, **p < 0.01, and ***p < 0.001. *p* > 0.05 indicated the absence of significant differences between the two groups and was marked as not significant (ns).

## Author Contributions

F. L., Y. X., Y. L. and Y. Z. were responsible for the preparation and characterization experiments of biomaterials. F. L., L. X., Y. X., H. K. and Z. Z. were responsible for cell experiments and verification of molecular mechanisms. Y. Z., J. C. and N. Z. were in charge of animal experiments. X. Y. and W. C. provided technical support and theoretical guidance for the project. F. L. was responsible for writing the manuscript, and W. C. made revisions to the manuscript.

## Conflicts of Interest

The authors declare no conflict of interest.

## Ethical Approval Statement

The animal experiments were approved by the Animal Research Committee of the Second Affiliated Hospital of Zhejiang University School of Medicine (AIRB‐2024‐0059).

## Supporting information




**Supporting File**: exp270195‐sup‐0001‐SuppMat.pdf.

## Data Availability

The data that support the findings of this study are available from the corresponding author upon reasonable request.

## References

[1] V. S. Ndaw , D. Abebayehu , A. J. Spence , et al., "“TGF‐β1 Suppresses IL‐33–Induced Mast Cell Function,” The Journal of Immunology 199 (2017): 866. 28637902 10.4049/jimmunol.1601983 PMC5538185

[2] Y. L. Xu , M. H. Zhang , W. Guo , et al., “MicroRNA‐19 Restores Vascular Endothelial Cell Function in Lower Limb Ischemia‐Reperfusion Injury Through the KLF10‐Dependent TGF‐β1/Smad Signaling Pathway in Rats,” Journal of Cellular Biochemistry 119 (2018): 9303–9315, 10.1002/jcb.27207. 29953651

[3] K. Plinta , A. Plewka , M. Wojcik‐Pedziwiatr , N. Zmarzly , M. Rudzinski , and M. Rudzinska‐Bar , “Potentially Detrimental Effects of Hyperosmolality in Patients Treated for Traumatic Brain Injury,” Journal of Clinical Medicine 10 (2021): 4141.34575255

[4] E. Jun , A. Y. Song , J. W. Choi , et al., “Progressive Impairment of NK Cell Cytotoxic Degranulation Is Associated With TGF‐β1 Deregulation and Disease Progression in Pancreatic Cancer,” Frontiers in Immunology 10 (2019): 1354, 10.3389/fimmu.2019.01354. 31281312 PMC6598013

[5] Y. Wang , Z. Jin , S. Jia , P. Shen , Y. Yang , and Y. Huang , “Mechanical Stress Protects Against Chondrocyte Pyroptosis Through TGF‐β1‐Mediated Activation of Smad2/3 and Inhibition of the NF‐κB Signaling Pathway in an Osteoarthritis Model,” Biomedicine & Pharmacotherapy 159 (2023): 114216, 10.1016/j.biopha.2023.114216. 36634591

[6] C. Liu , P. Li , X. Ao , et al., “Clusterin Negatively Modulates Mechanical Stress‐Mediated Ligamentum Flavum Hypertrophy Through TGF‐β1 Signaling,” Experimental & Molecular Medicine 54 (2022): 1549–1562, 10.1038/s12276-022-00849-2. 36131026 PMC9534863

[7] Y.‐Z. Huang , L. Zhao , Y. Zhu , et al., “Interrupting TGF‐β1/CCN2/Integrin‐α5β1 Signaling Alleviates High Mechanical‐Stress Caused Chondrocyte Fibrosis,” European Review for Medical and Pharmacological Sciences 25 (2021): 1233–1241. 33629293 10.26355/eurrev_202102_24827

[8] L. Marshall , A. Tarakanova , P. Szarek , and D. M. Pierce , “Cartilage and Collagen Mechanics Under Large‐Strain Shear Within in Vivo and at Supraphysiogical Temperatures,” Journal of the Mechanical Behavior of Biomedical Materials 103 (2020): 103595, 10.1016/j.jmbbm.2019.103595. 32090923

[9] A. P. Ronkainen , P. Tanska , J. M. Fick , W. Herzog , and R. K. Korhonen , “Interrelationship of Cartilage Composition and Chondrocyte Mechanics After a Partial Meniscectomy in the Rabbit Knee Joint—Experimental and Numerical Analysis,” Journal of Biomechanics 83 (2019): 65–75, 10.1016/j.jbiomech.2018.11.024. 30501912

[10] S. Lin , H. Li , B. Wu , et al., “TGF‐β1 Regulates Chondrocyte Proliferation and Extracellular Matrix Synthesis via circPhf21a‐Vegfa Axis in Osteoarthritis,” Cell Communication and Signaling 20 (2022): 75, 10.1186/s12964-022-00881-9. 35637489 PMC9150374

[11] X. Che , X. Jin , N. R. Park , et al., “Cbfβ Is a Novel Modulator Against Osteoarthritis by Maintaining Articular Cartilage Homeostasis Through TGF‐β Signaling,” Cells 12 (2023): 1064. 37048137 10.3390/cells12071064 PMC10093452

[12] G. Zhen , Q. Guo , Y. Li , et al., “Mechanical Stress Determines the Configuration of TGFβ Activation in Articular Cartilage,” Nature Communications 12 (2021): 1706, 10.1038/s41467-021-21948-0. PMC7969741 33731712

[13] F. Lin , Y. Zhuang , L. Xiang , et al., “Localization of Lesion Cells and Targeted Mitochondria Via Embedded Hydrogel Microsphere using Heat Transfer Microneedles,” Advanced Functional Materials (2023): 33 2212730.

[14] F. Lin , Y. Li , and W. Cui , “Injectable Hydrogel Microspheres in Cartilage Repair,” Biomedical Technology 1 (2023): 18–29, 10.1016/j.bmt.2022.11.002.

[15] L. Chen , J. Zhang , J. Wang , J. Lin , X. Luo , and W. Cui , “Lithiation Enhances Electrocatalytic Iodine Conversion and Polyiodide Confinement in Iodine Host for Zinc–Iodine Batteries,” Advanced Functional Materials 33 (2023): 2304811.

[16] Z. Luo , Y. Wang , Y. Xu , J. Wang , and Y. Yu , “Modification and Crosslinking Strategies for Hyaluronic Acid‐Based Hydrogel Biomaterials,” Smart Medicine 2 (2023): e20230029, 10.1002/SMMD.20230029. 39188300 PMC11235888

[17] F. Lin , Z. Wang , L. Xiang , L. Deng , and W. Cui , “Inorganic Electron Transport Materials in Perovskite Solar Cells,” Advanced Functional Materials 31 (2021): 2008300.

[18] H. Zhang , X. Kong , Y. Tang , and W. Lin , “Hydrogen Sulfide Triggered Charge‐Reversal Micelles for Cancer‐Targeted Drug Delivery and Imaging,” ACS Applied Materials & Interfaces 8 (2016): 16227–16239, 10.1021/acsami.6b03254. 27280335

[19] C. Y. Shen , Q. R. Zhou , X. Wu , et al., “Accelerating cartilage regeneration with DNA‐SF hydrogel sustained release system‐based cartilage organoids,” Military Medical Research 12 (2025): 39. 40722047 10.1186/s40779-025-00625-z PMC12302690

[20] C. Shen , Z. Zhou , R. Li , et al., “Silk Fibroin‐Based Hydrogels for Cartilage Organoids in Osteoarthritis Treatment,” Theranostics 15 (2025): 560–584, 10.7150/thno.103491. 39744693 PMC11671376

[21] X. Li , S. Sheng , G. Li , et al., “Research Progress in Hydrogels for Cartilage Organoids,” Advanced Healthcare Materials 13 (2024): e2400431, 10.1002/adhm.202400431. 38768997

[22] D. Zheng , W. Chen , H. Ruan , et al., “Metformin‐hydrogel with glucose responsiveness for chronic inflammatory suppression,” Chemical Engineering Journal 428 (2022): 131064.

[23] Z. Fu , Y. Lai , Y. Zhuang , and F. Lin , “Injectable Heat‐Sensitive Nanocomposite Hydrogel for Regulating Gene Expression in the Treatment of Alcohol‐Induced Osteonecrosis of the Femoral Head,” APL Bioengineering 7 (2023): 16107, 10.1063/5.0130711. PMC9862308 36691581

[24] F. Lin , Z. Wang , L. Xiang , et al., “Simultaneous Lattice Engineering and Defect Control via Cadmium Incorporation for High‐Performance Inorganic Perovskite Solar Cells,” Advanced Science 9 (2022): 2204486. 36344454 10.1002/advs.202204486 PMC9798970

