# Supplementary material for: Matching TGF‐β1 Activation With Stress‐Responsive Charge‐Reversal Hydrogel Microspheres for the Treatment of Osteoarthritis
Source: Exploration (Beijing). 2026 Jun 9:20250363. Online ahead of print. doi: 10.1002/EXP.20250363 (PMC13394782; doi:10.1002/EXP.20250363)
Supplement: Supplementary file 1 — Supporting File: exp270195‐sup‐0001‐SuppMat.pdf. [file EXP2-9999-0-s001.pdf]

Supporting Information

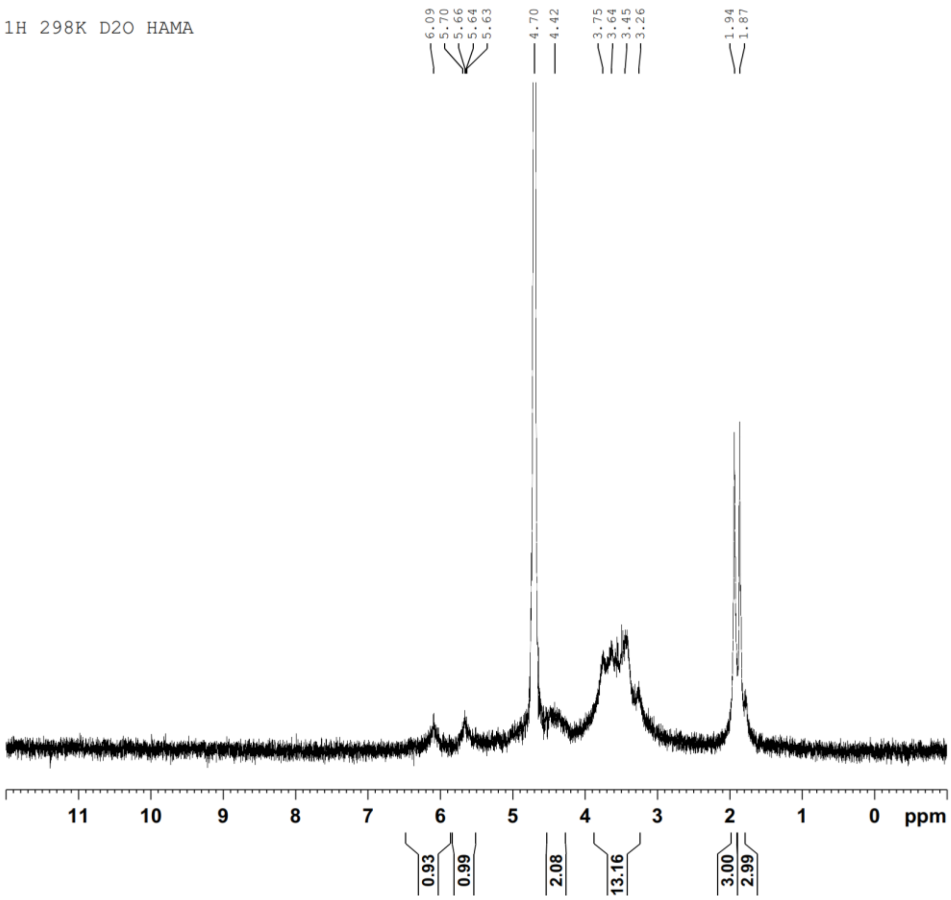

Figure S1. The ^{1}H NMR map and integral calculation of HAMA.

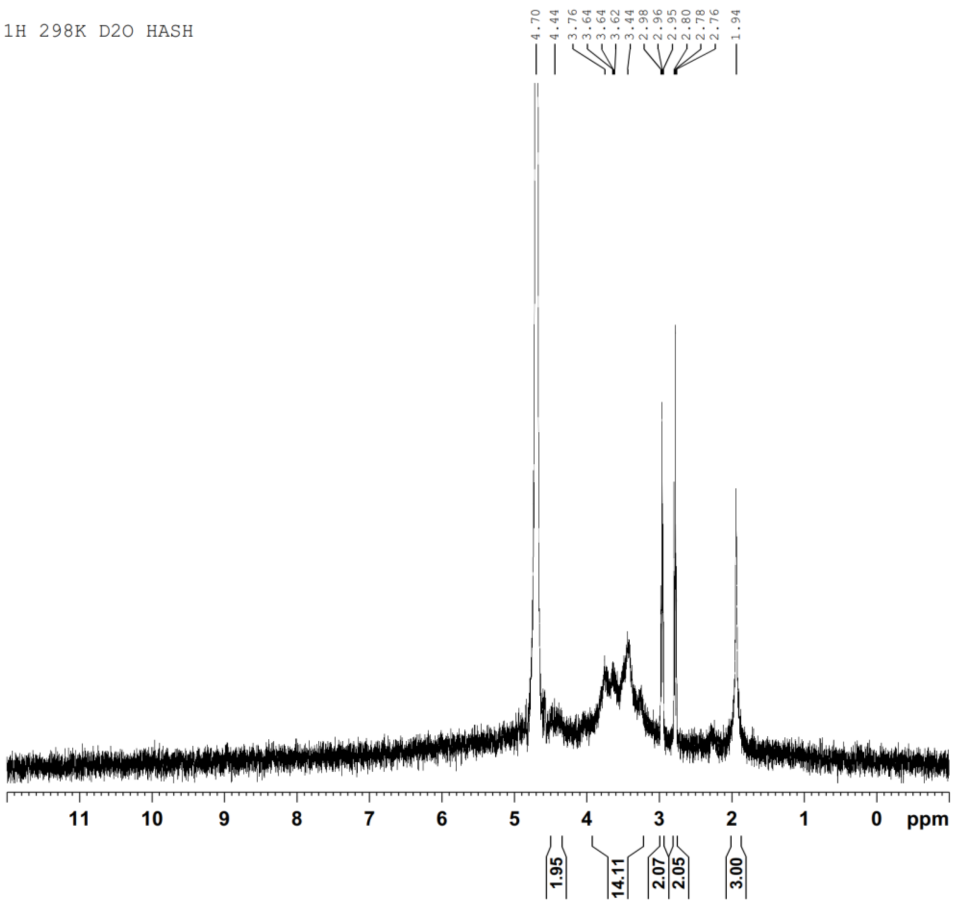

Figure S2. The ^{1}H NMR map and integral calculation of HASH.

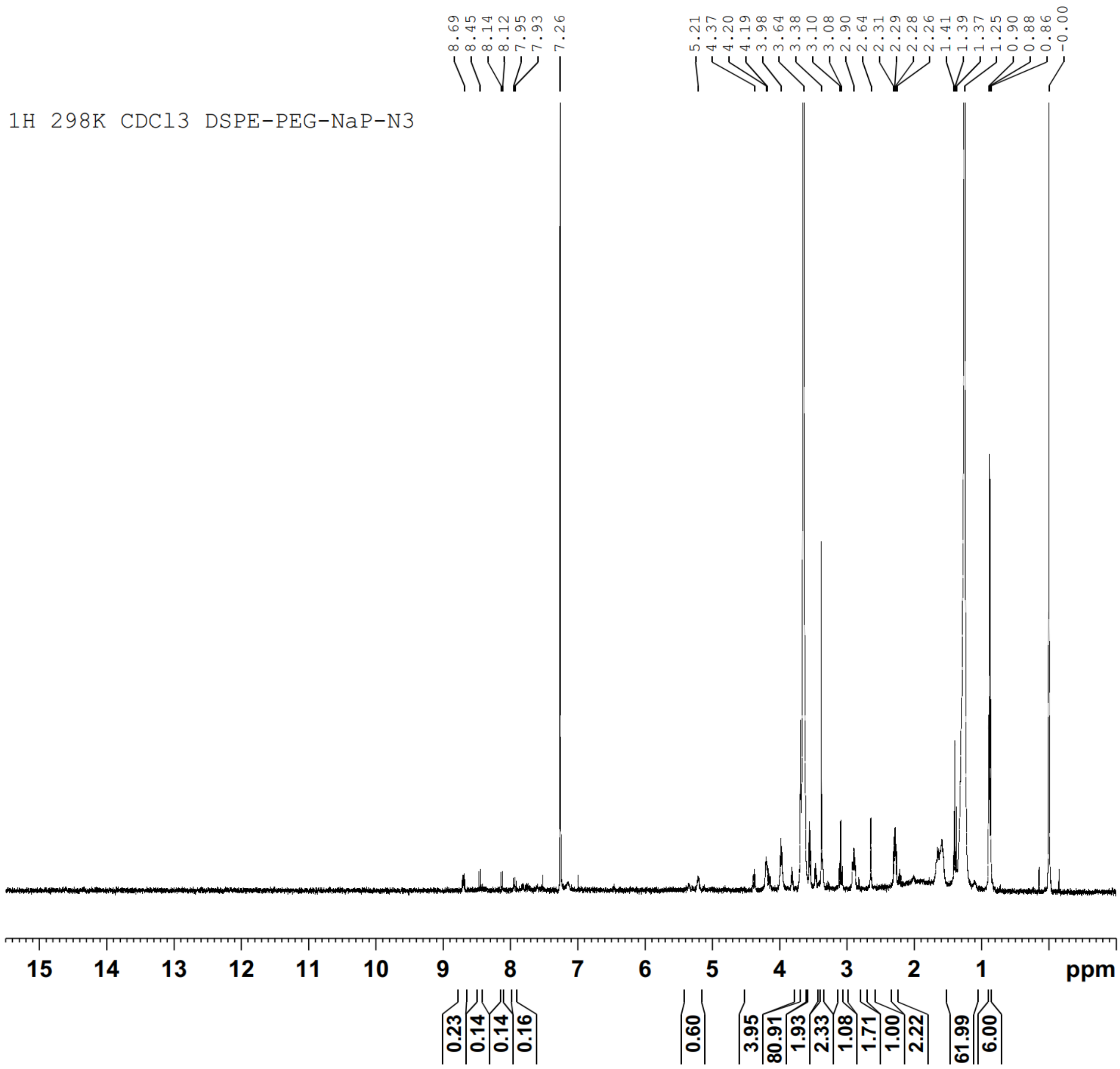

Figure S3. The ^{1}H NMR map and integral calculation of DSPE-PEG-Nap-N_3.

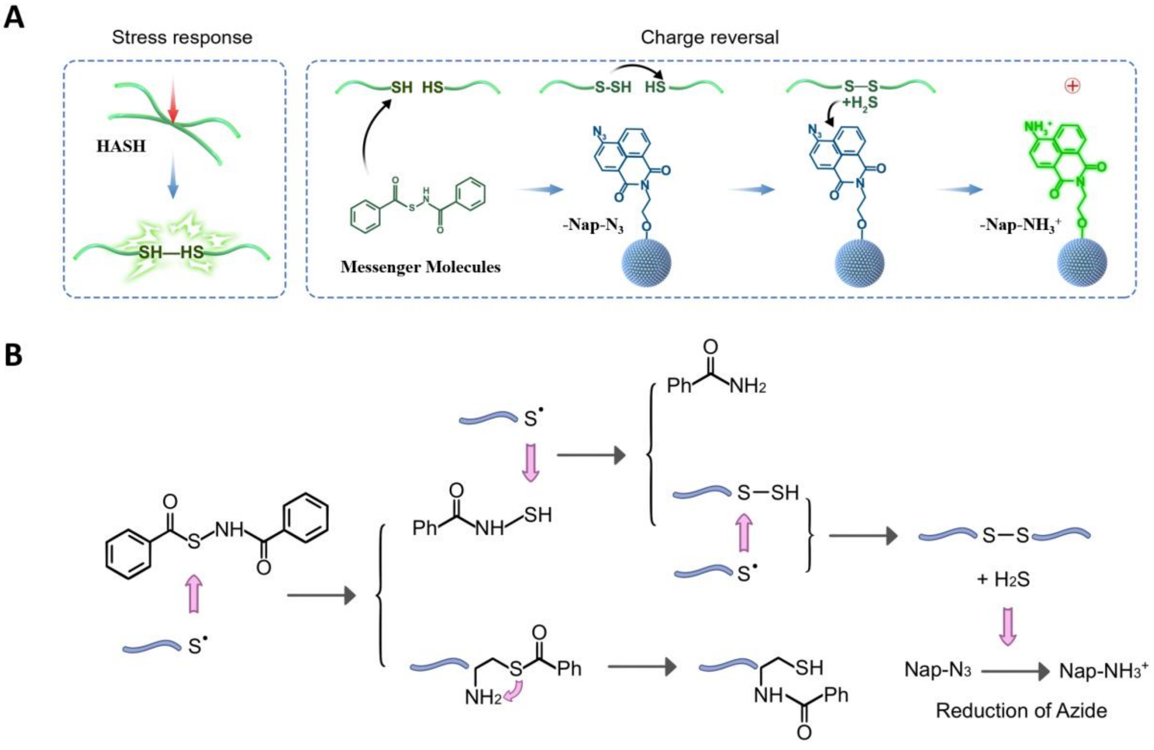

Figure S4. A) Schematic diagram of the stress-responsive charge-reversal mechanism. B） The detailed chemical reaction for the stress-responsive charge-reversal mechanism.

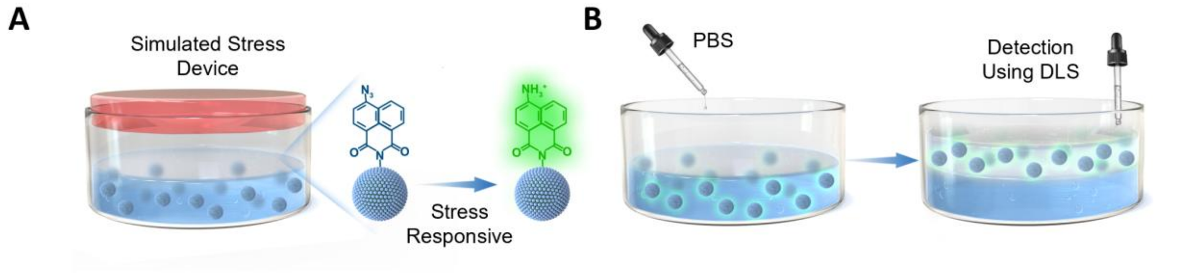

Figure S5. A) Schematic diagram of the charge-reversal experiment of the nanoliposomes. B) Schematic diagram of the experiment to detect the zeta potential of charge-reversal nanoliposomes.

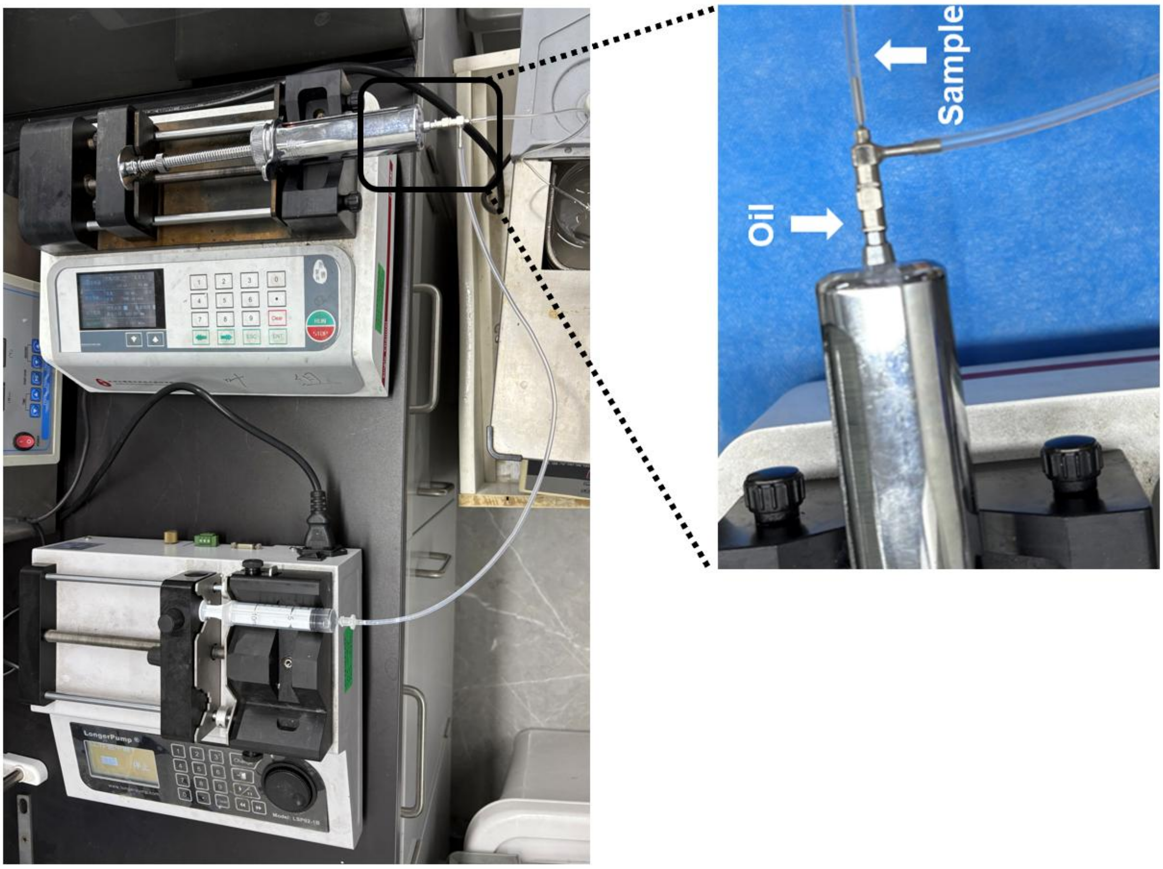

Figure S6. The photograph of the microfluidic device.

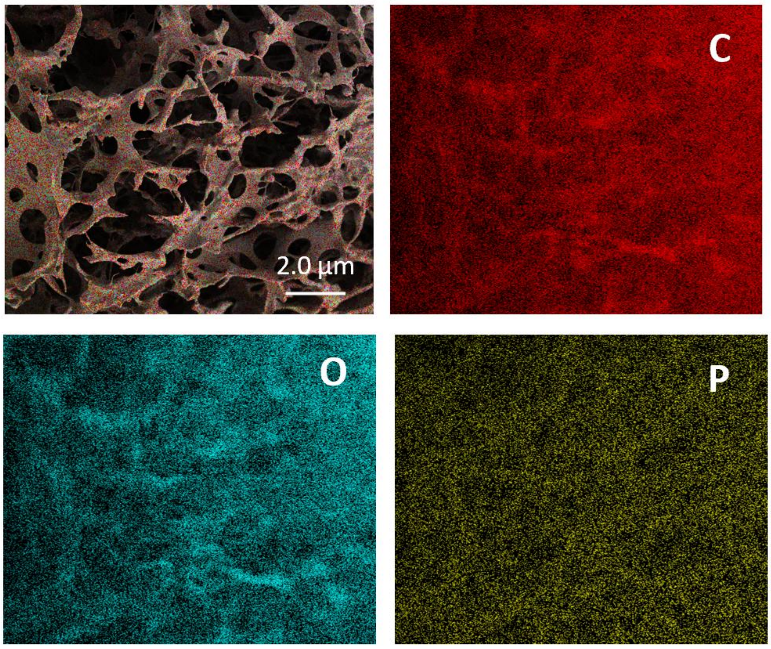

Figure S7. Elemental energy spectrum detection of the hydrogel microsphere system.

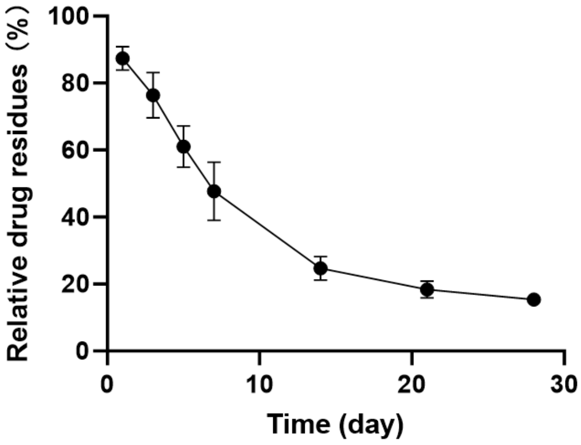

Figure S8. Release curve of drug LSKL in the aqueous coagulation microsphere system.

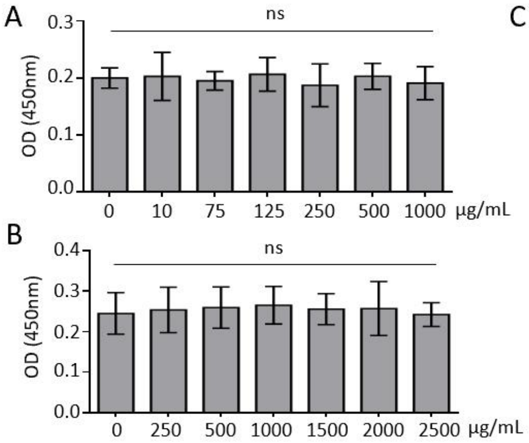

Figure S9. Biocompatibility test of hydrogel microspheres system. A) Effect of nanoparticles at different concentrations on chondrocyte proliferation. B) Effects of different concentrations of hydrogel microspheres on the proliferation of chondrocytes. C) Results of live/dead staining of chondrocytes by the highest concentration nanoparticle and hydrogel microsphere systems. (ns: non-significant)

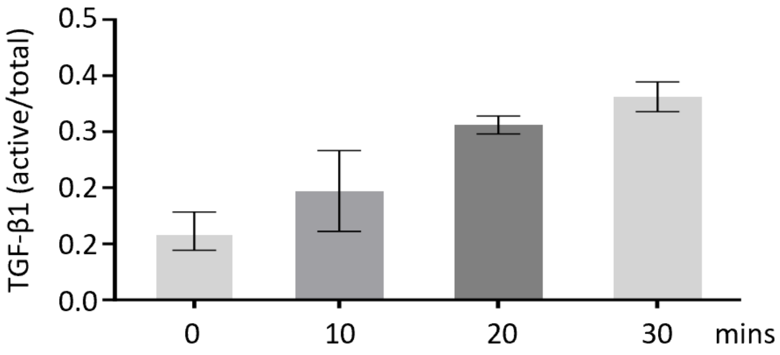

Figure S10. The dynamic changes of the TGF-β1 activation ratio at different time points.

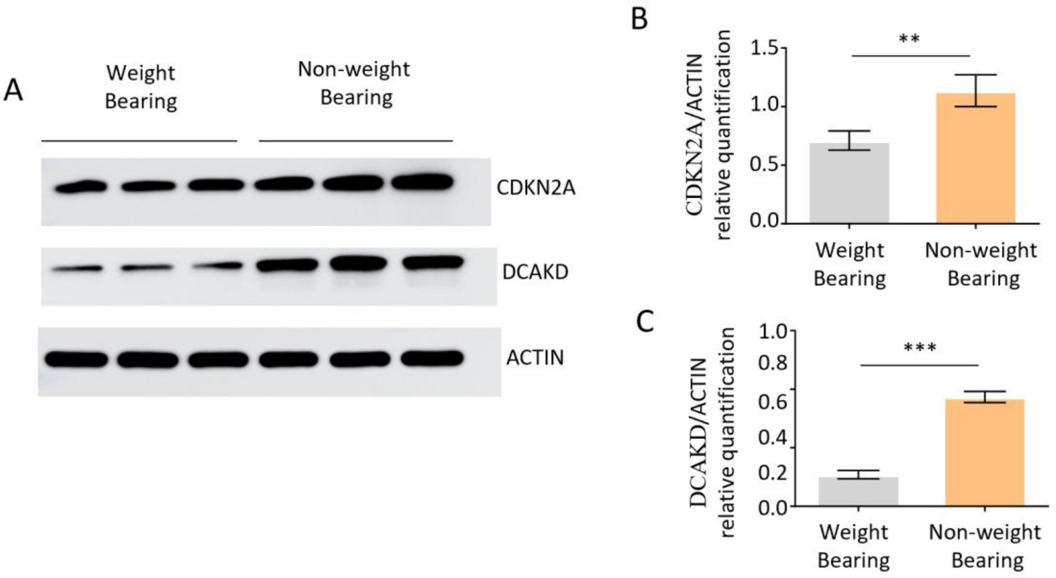

Figure S11. Verification of the phenomenon of differential expression of mitochondrial-related genes. A) Detection of CDKN2A and DCAKD expression in chondrocytes in different experimental groups. B) Quantitative analysis of western blot results (n = 3). (** $P < 0.01$, *** $P < 0.001$)

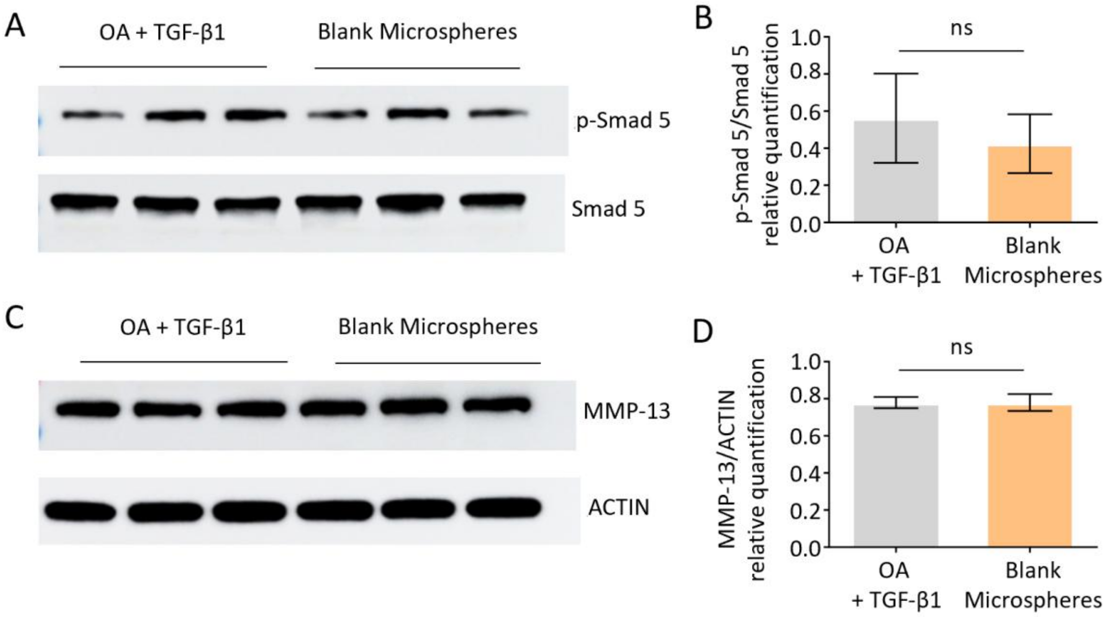

Figure S12. The therapeutic effect of the blank microspheres. A) Detection of p-Smad 5 and Smad 5 expression in chondrocytes in different experimental groups. B) Quantitative analysis of western blot results (n = 3). C) Detection of MMP-13 expression in chondrocytes in different experimental groups. D) Quantitative analysis of western blot results (n = 3).(ns, not significant)

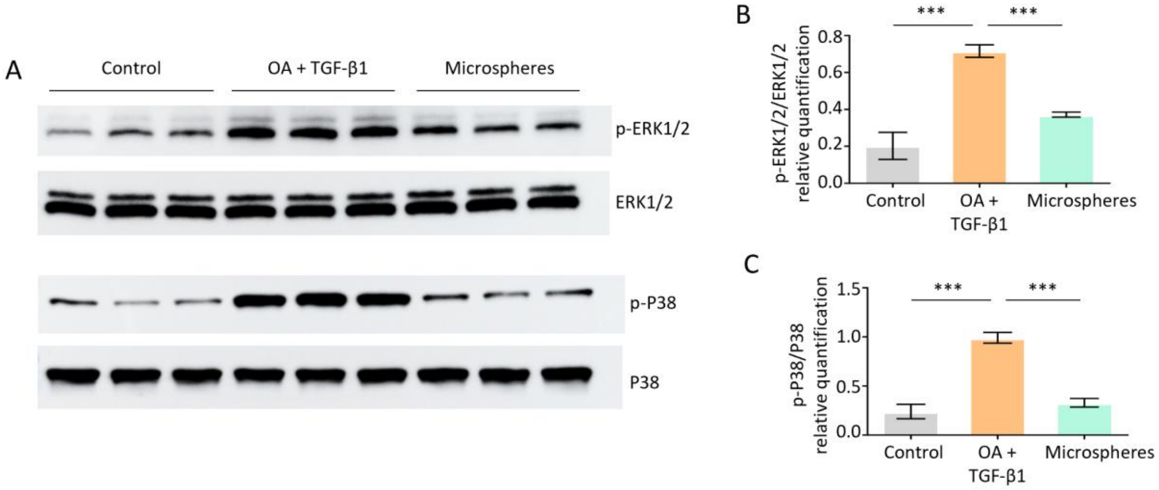

Figure S13. The influence of TGF-β1 on the MARK signaling pathway. A) Detection of p-ERK1/2, ERK1/2, p-P38 and P38 expression in chondrocytes in different experimental groups. B-C) Quantitative analysis of western blot results (n = 3). (*** $P < 0.001$)

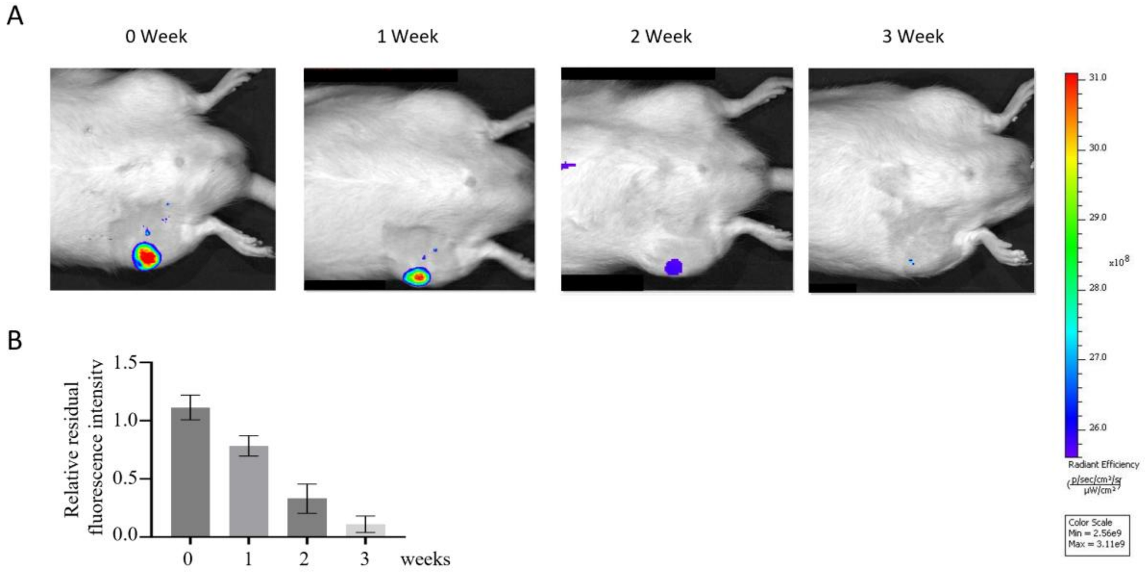

Figure S14. The *in vivo* fluorescence residue experiment in SD rats. A) Fluorescence residue experiment of hydrogel microspheres. B) Quantitative analysis of residual fluorescence values.

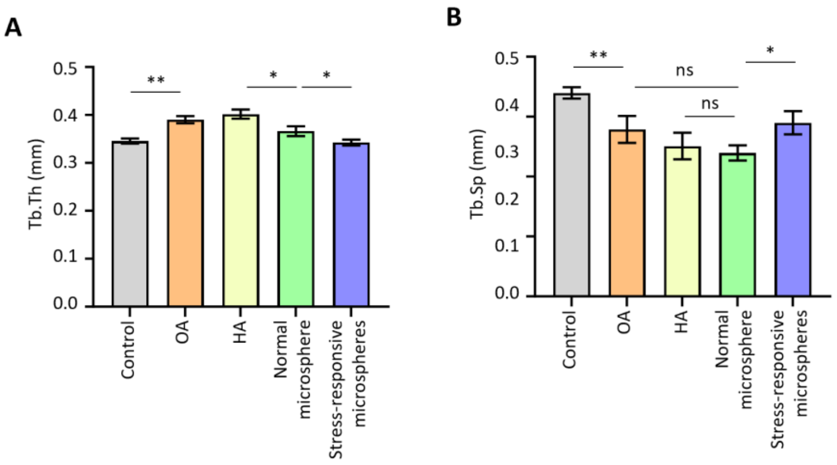

Figure S15. A) Quantitative analysis of the bone trabecular thickness (Tb.Th) values (n = 3). B) Quantitative analysis of the trabecular separation (Tb.Sp) (n = 3). (ns, not significant, * $P < 0.05$, ** $P < 0.01$)

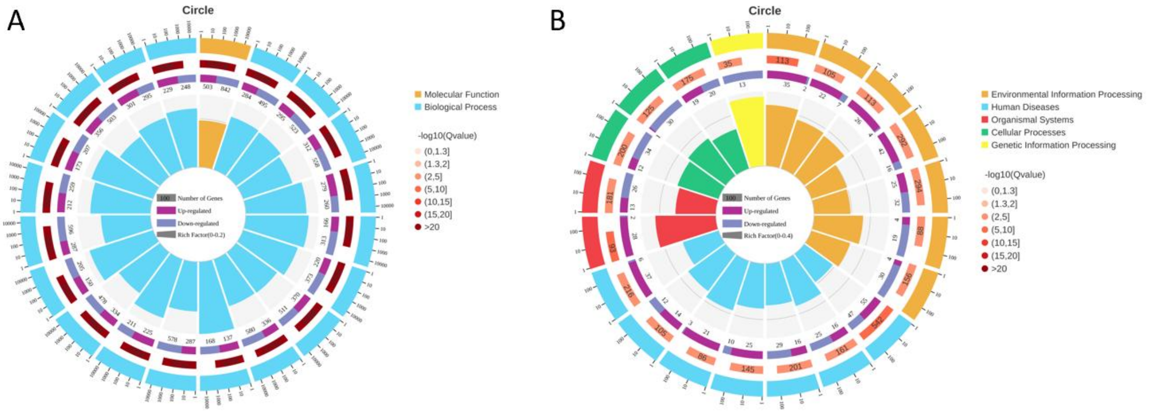

Figure S16. Enrichment results of differentially expressed genes. A) the Gene Ontology (GO) items with the highest enrichment scores. B) The Kyoto Encyclopedia of Genes and Genomes (KEGG) items with the highest enrichment scores.

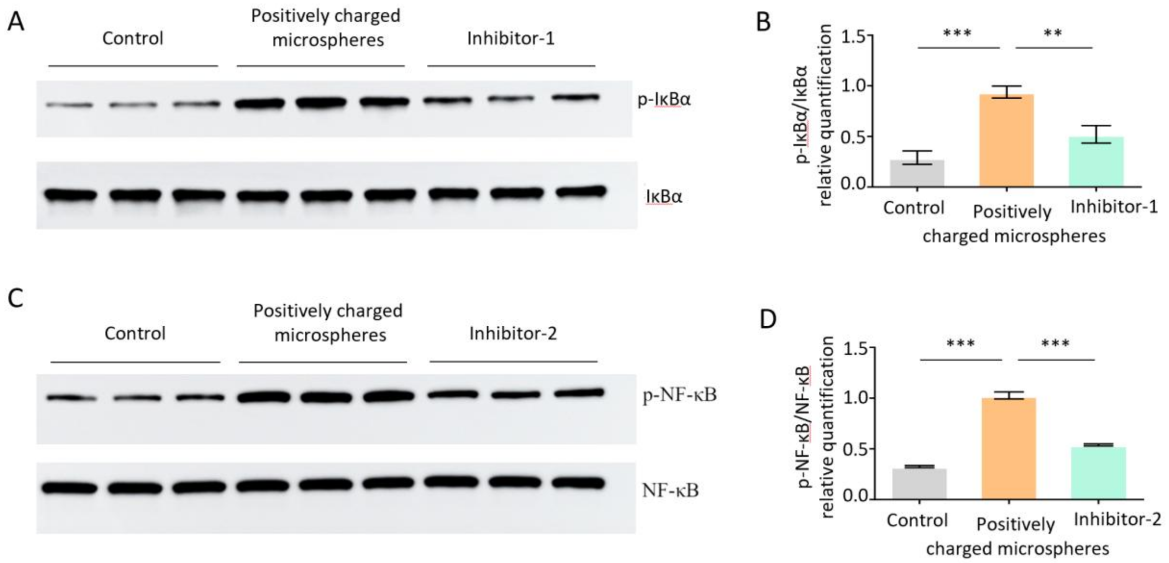

Figure S17. The influence of microspheres on the TNF signaling pathway and the NF-κB signaling pathway. A) Detection of p-IκBα and IκBα expression in chondrocytes in different experimental groups. B) Quantitative analysis of western blot results (n = 3). C) Detection of p-NF-κB and NF-κB expression in chondrocytes in different experimental groups. D) Quantitative analysis of western blot results (n = 3).(Inhibitor-1: maslinic acid; Inhibitor-2: NF-κB-IN-1), (** $P < 0.01$, *** $P < 0.001$)

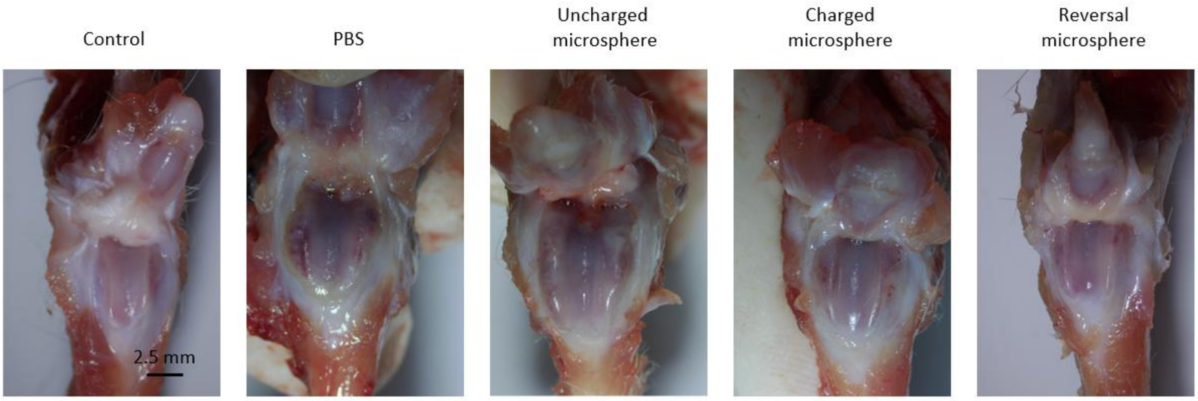

Figure S18. The photographs of rat knee cartilage from different experimental groups.

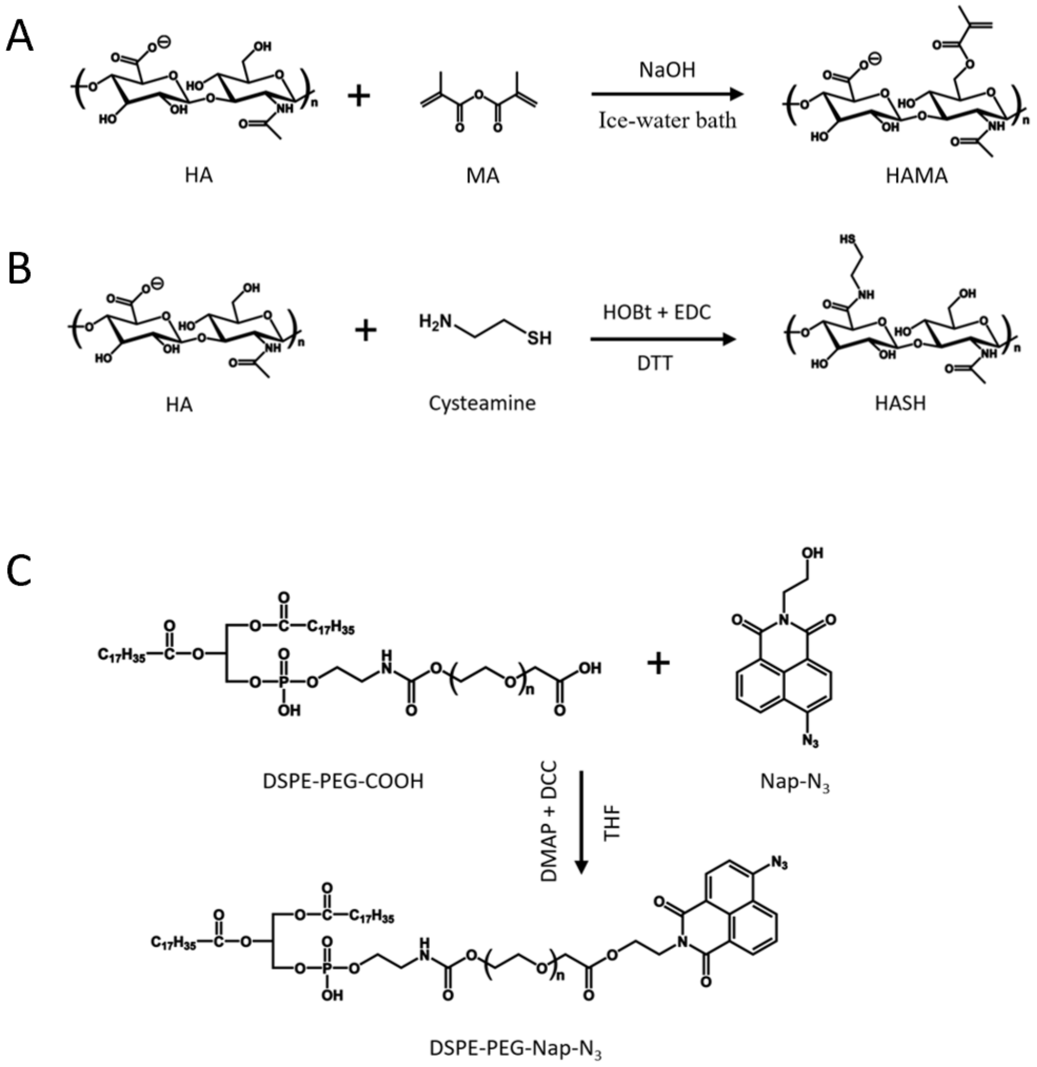

Figure S19. A) Schematic diagram of HAMA polymer synthesis. B) Schematic diagram of HASH polymer synthesis. C) Schematic diagram of DSPE-PEG-Nap-N_3 polymer synthesis.

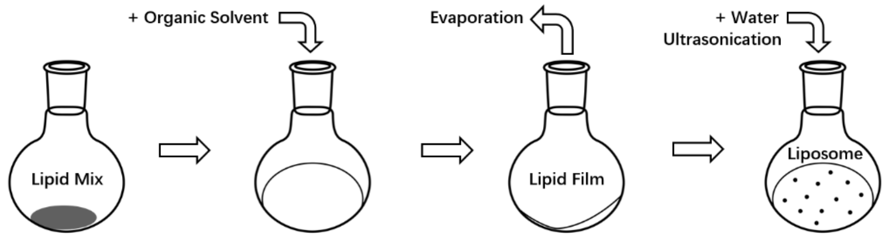

Figure S20. Schematic diagram of preparing liposomes by thin-film hydration method.
